# Whole-genome sequencing-based analyses of drug-resistant *Mycobacterium tuberculosis* from Taiwan

**DOI:** 10.1038/s41598-023-29652-3

**Published:** 2023-02-13

**Authors:** Yu-Xin Xiao, Kuang-Hung Liu, Wan-Hsuan Lin, Tai-Hua Chan, Ruwen Jou

**Affiliations:** 1 Tuberculosis Research Center, Taiwan Centers for Disease Control, Ministry of Health and Welfare, No. 161, Kun-Yang Street, Taipei, 11561 Taiwan, R.O.C.; 2 Reference Laboratory of Mycobacteriology, Centers for Disease Control, Ministry of Health and Welfare, Taipei, Taiwan, R.O.C.

**Keywords:** Genetics, Microbiology

## Abstract

Drug-resistant tuberculosis (DR-TB) posed challenges to global TB control. Whole-genome sequencing (WGS) is recommended for predicting drug resistance to guide DR-TB treatment and management. Nevertheless, data are lacking in Taiwan. Phenotypic drug susceptibility testing (DST) of 12 anti-TB drugs was performed for 200 *Mycobacterium tuberculosis* isolates. WGS was performed using the Illumina platform. Drug resistance profiles and lineages were predicted in silico using the Total Genotyping Solution for TB (TGS-TB). Using the phenotypic DST results as a reference, WGS-based prediction demonstrated high concordance rates of isoniazid (95.0%), rifampicin (RIF) (98.0%), pyrazinamide (98.5%) and fluoroquinolones (FQs) (99.5%) and 96.0% to 99.5% for second-line injectable drugs (SLIDs); whereas, lower concordance rates of ethambutol (87.5%), streptomycin (88.0%) and ethionamide (84.0%). Furthermore, minimum inhibitory concentrations confirmed that RIF *rpoB* S450L, FQs *gyrA* D94G and SLIDs *rrs* a1401g conferred high resistance levels. Besides, we identified lineage-associated mutations in lineage 1 (*rpoB* H445Y and *fabG1* c-15t) and predominant lineage 2 (*rpoB* S450L and *rpsL* K43R). The WGS-based prediction of drug resistance is highly concordant with phenotypic DST results and can provide comprehensive genetic information to guide DR-TB precision therapies in Taiwan.

## Introduction

According to the World Health Organization (WHO) global tuberculosis (TB) report, an estimated of 5.8 million new cases and 157,903 rifampicin (RIF)/multidrug-resistant (RR/MDR) TB cases in 2020^1^. Nevertheless, approximately 33.3% of RR/MDR-TB cases were detected, and 59.0% are successfully treated^1^. Closing the gap in the detection of drug-resistance (DR)-TB cases requires universal and timely drug susceptibility testing (DST).

Conventional culture-based DST was the gold standard for DR-TB diagnoses, but it is time-consuming and labor intensive. Rapid molecular tests, such as the GeneXpert and line probe assays, have been adopted as diagnostic alternatives for *Mycobacterium tuberculosis* detection and DR prediction^2^. Nevertheless, these assays could only detect limited number of mutations and show low sensitivity for hetero-resistant strains with variant frequencies below 5–50%^3^.

Whole-genome sequencing (WGS) enables the identification of single-nucleotide polymorphisms (SNPs) and insertions and deletions (indels) in loci associated with drug resistance and are proven to have higher accuracy than phenotypic DST^4^. Since noncanonical mutations in known or unknown genes or other mechanisms still need to be identified in 10–40% of DR isolates, WGS can comprehensively identify drug resistance-associated genes to indicate drug susceptibility for clinical decision making^5^. Several bioinformatics tools have been developed for inferring drug resistance from WGS data, including KvarQ, PhyResSE, CASTB, Mykrobe, TB Profiler, and Total Genotyping Solution for TB (TGS-TB)^6–10^. The TGS-TB emphasis particularly on Beijing genotype *M. tuberculosis*, which is predominant in East Asia where Taiwan is located^10^. Previous study reported that performance of TGS-TB in predicting resistance to first-line drugs is comparable to other tools^6^. Nevertheless, data are lacking in Taiwan.

To strengthen DR-TB diagnosis, we report the performance of WGS with the TGS-TB to analyze (sub)lineages and prediction of *M. tuberculosis* drug resistance.

## Materials and methods

### Study population

We collected 200 isolates from approximately 30% of RR-/MDR-TB confirmed cases during 2013–2016. One *M. tuberculosis* isolate from each case was analyzed. Cultivation and processing of *M. tuberculosis* isolates were performed in a certified biosafety level 3 laboratory. Isolates were obtained by processing specimens with standard N-acetyl-L-cysteine (NALC)-NaOH method^11^, then inoculated onto Bactec MGIT 960 system. Information on the study cases was obtained from the National TB Registry.

### Ethics statement

This study was approved by the Institutional Review Board of the Taiwan Centers for Disease Control (TwCDC IRB No. 106211). All methods were performed in accordance with the relevant guidelines and regulations. The study analyzed only archived isolates, and the need for the written informed consent of the participants was waived.

### Phenotypic drug susceptibility testing

DST was conducted using the agar proportion method (APM) with 7H10 and 7H11 medium (Becton, Dickinson and Company, Spark, MD, USA). Drug resistance was defined as the growth of 1% of colonies in drug-containing medium. The critical concentrations of the tested drugs in 7H10 medium were as follows: rifampicin (RIF), 1 μg/mL; isoniazid (INH), 0.2 μg/mL; ethambutol (EMB), 5 μg/mL; streptomycin (STR), 2 μg/mL; ofloxacin (OFX), 2 μg/mL; and moxifloxacin (MFX), 0.5 μg/mL. The critical concentrations of the tested drugs in 7H11 medium were as follows: kanamycin (KM), 6 μg/mL; amikacin (AMK), 6 μg/mL; capreomycin (CM), 10 μg/mL; ethionamide (ETO), 10 μg/mL; and para-aminosalicylic acid (PAS), 8.0 μg/mL. Resistance to pyrazinamide (PZA) at 100 μg/mL was tested using Bactec MGIT 960 as described previously^12^. Inocula were cultured in a 37 °C incubator for 3 weeks. The DST results were categorized as resistant or susceptible, and the H37Rv (ATCC 27294) strain was used as the control. MDR is defined as an *M. tuberculosis* isolate resistant to at least INH and RIF. Pre-XDR is defined as an MDR isolate resistant to either fluoroquinolones (FQs) or second-line injectable drugs (SLIDs)^13^. XDR is defined as an MDR isolate resistant to both FQs and SLIDs^14^.

### Minimum inhibitory concentration (MIC) testing

Phenotypic MIC testing was performed using the Sensititre™ *Mycobacterium tuberculosis* MYCOTB assay (Thermo Scientific™, TREK Diagnostic Systems, United Kingdom) following the manufacturer’s instructions. The 96-well microtiter plates of the assay containing RIF, INH, EMB, STR, rifabutin (RFB), OFX, MFX, KM, AMK, ETO, PAS and cycloserine (CS). The H37Rv (ATCC 27294) strain was used as the control. The plates were incubated at 37 °C for 2 weeks. The MIC values were recorded by 2 independent readers and a third reading was sought if a discrepant reading was found.

### Whole-genome sequencing

Genomic DNA was extracted using the Gentra Puregene Yeast/Bact. Kit (QIAGEN GmbH, Hilden, Germany) following the manufacturer’s protocol, and was quantified using a Qubit 2.0 fluorometer (ThermoFisher Scientific, Waltham, MA, USA). WGS was performed as previously described^15^. Paired-end libraries were prepared using the QIAseq FX DNA Library Kit (QIAGEN GmbH, Hilden, Germany) according to the manufacturer’s protocol. The average fragment size (500–600 bp) of the DNA libraries was estimated by 2% agarose gel electrophoresis. Then, the fragments were eluted using the Wizard SV Gel and PCR Clean-Up System (Promega Corporation, Madison, WI, USA). The 24 purified DNA libraries were pooled (11 pM) were sequenced on an Illumina MiSeq system (Illumina, Inc., San Diego, CA, USA) with the MiSeq Reagent Kit ver. 3 (600 cycles).

### Bioinformatic analysis

Sequence reads were checked using FastQC (www.bioinformatics.babraham. ac.uk/projects/fastqc/) for initial assessment of data quality. Drug resistance prediction and lineage analysis were performed using the web-based TGS-TB v2^10^. The following drug-resistance associated genes were predicted: RIF (*rpoB*, *rpoC*), INH (*katG*, *fabG1*, *ahpC*, *inhA*), EMB (*embA*, *embB*, *embC*), PZA (*pncA*), FQs (*gyrA*, *gyrB*), STR (*rpsL*, *rrs*, *gid*), SLIDs (*rrs*, *eis*), ETO (*ethA*, *ethR*), and PAS (*folC, thyA*). A phylogenetic tree was constructed from reliable SNPs with respect to H37Rv (NC_000962.3) using the maximum likelihood method with the Tamura-Nei model in MEGA 7.0^16^; 1,000 bootstrap replicates were conducted. The tree was annotated and visualized using iTOL v6 (https://itol.embl.de)^17^.

### Statistical analysis

Descriptive statistics of demographics and clinical characteristics of study cases were presented as proportions. Odds ratios (ORs) and 95% confidence intervals (CIs) were calculated to estimate the correlation between the lineages and variables. The chi-squared test or Fisher’s exact test (when expected cell size < 5) was used for the univariate analysis of categorical variables. Statistical significance was considered as *P* < 0.05.

## Results

### Characteristics of the study population

**Figure 1:**
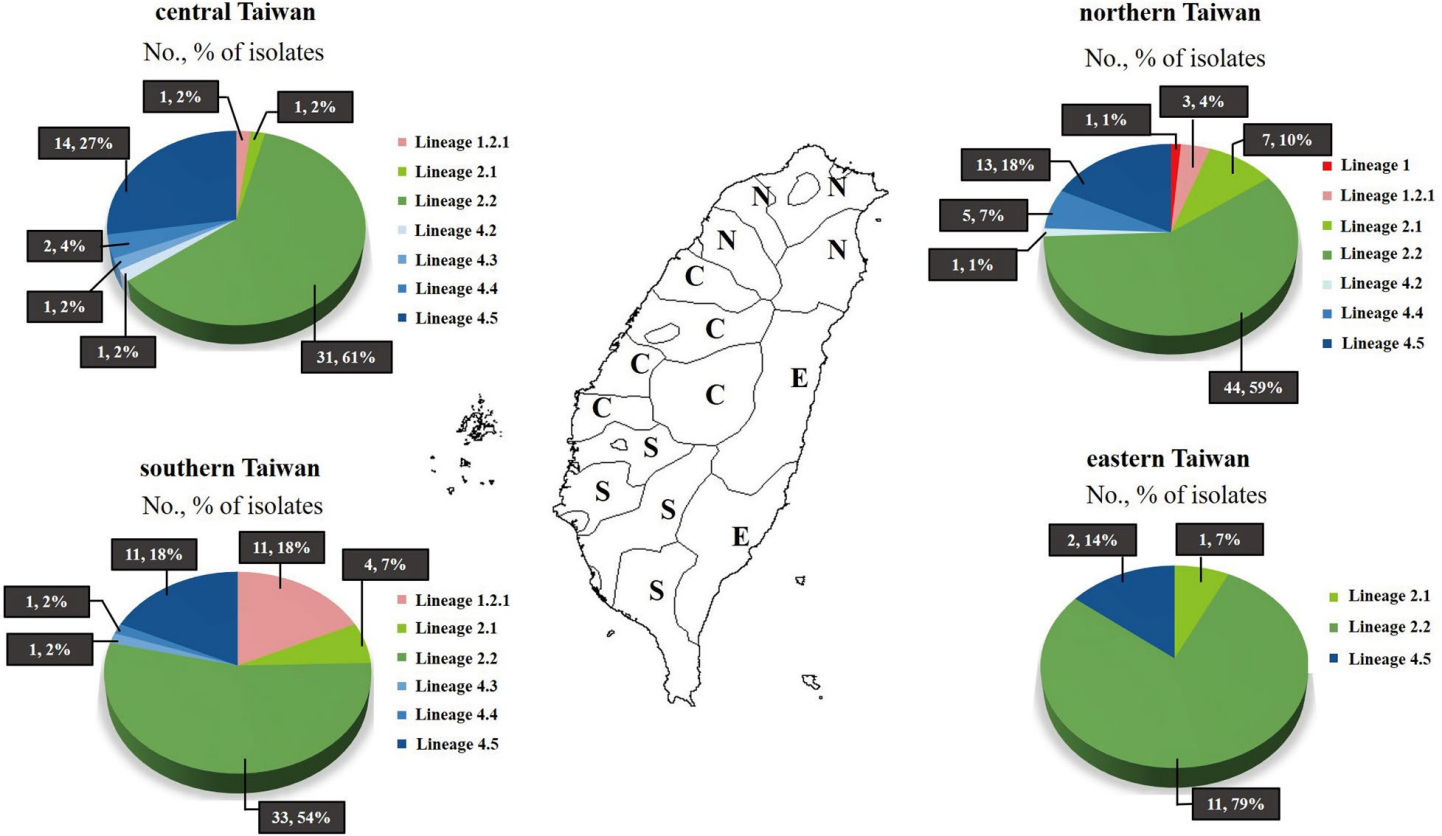
Geographic distribution of lineages and sublineages of drug-resistant *M. tuberculosis* isolates in Taiwan. The distribution of each phenotype in each district is represented in the corresponding pie chart as indicated. Abbreviations: N, northern Taiwan; E, eastern Taiwan; C, central Taiwan; S, southern Taiwan.

Among the 200 DR-TB cases, 146 (73.0%) cases were male, the median age was 66 (interquartile range = 55–78) years, 165 (82.5%) were new cases and 182 (91.0%) cases showed pulmonary TB (Table 1). The majority of DR-TB cases came from northern (74, 37.0%) Taiwan. Among the 200 DR *M. tuberculosis* isolates, the predominant lineages were lineage 2 East Asian (132, 66.0%) and lineage 4 Euro-American (52, 26.0%) (Table 1). Sublineage 2.2 isolates were isolated from eastern (11, 79.0%), central (31, 61.0%), northern (44, 59.0%) and southern (33, 54.0%) Taiwan. The sublineage 1.2.1 isolates mainly came from southern (11, 18.0%) Taiwan (Fig. 1).

**Table 1:** Demographics and characteristics of the 200 study cases.

Characteristic	Case no. (%)
Sex	
Male	146 (73.0)
Female	54 (27.0)
Age	
< 25	2 (1.0)
25–44	26 (13.0)
44–54	22 (11.0)
55–64	44 (22.0)
≥ 65	106 (53.0)
Region	
Northern	74 (37.0)
Southern	61 (31.0)
Central	51 (25.0)
Eastern	14 (7.0)
Case category	
New	165 (82.5)
Previously treated	35 (17.5)
Site of TB	
Pulmonary TB	182 (91.0)
Extrapulmonary TB	18 (9.0)
Acid fast bacillus smear	
Positive	114 (57.0)
Negative	86 (43.0)
Genotype	
Beijing	118 (59.0)
Non-Beijing	82 (41.0)
Lineage	
Lineage 1	16 (8.0)
1	1 (0.5)
1.2.1	15 (7.5)
Lineage 2	132 (66.0)
2.1	13 (6.5)
2.2	119 (59.5)
Lineage 4	52 (26.0)
4.2	2 (1.0)
4.3	2 (1.0)
4.4	8 (4.0)
4.5	40 (20.0)

### Drug resistance

#### Phenotypic drug resistance

Supplementary Table S1 showed the drug resistance profiles of the 200 isolates. Excluding two pan-susceptible isolates with disputed *rpoB* mutations and one STR mono-resistant isolate, the remaining isolates were RR (61, 31.0%) and MDR (136, 69.0%). Among 136 MDR isolates, 28 (20.6%) and 1 (0.7%) were pre-XDR and XDR, respectively (Table 2). The resistance rates to the tested drugs were as follows: RIF (197, 98.5%), INH (136, 68.0%), EMB (77, 38.5%), PZA (40, 20.0%), STR (60, 30.0%), FQs (22, 11.0%), KM (11, 5.5%), AMK (8, 4.0%), CM (6, 3.0%), ETO (34, 17.0%) and PAS (6, 3.0%) (Supplementary Table S2).

**Table 2:** Phenotypic drug resistance patterns of 197 RR and MDR/Pre-XDR/XDR *Mycobacterium tuberculosis* isolates.

DR patterns	RR (N = 61, 31.0%)		MDR (N = 136, 69.0%)	
	No. (%)	95% CI	No. (%)	95% CI
RIF	61 (100.0)	94.1–100.0	136 (100.0)	97.3–100.0
INH	0 (0.0)	0.0–5.9	136 (100.0)	97.3–100.0
EMB	1 (1.6)	0.3–8.7	76 (55.9)	47.5–64.0
PZA	2 (3.3)	0.9–11.2	38 (27.9)	21.1–36.0
STR	2 (3.3)	0.9–11.2	57 (41.9)	34.0–50.3
FQs	3 (4.9)	1.7–13.5	19 (14.0)	9.1–20.8
KM/AMK/CM	1 (1.6)	0.3–8.7	11 (8.1)	4.6–13.9
ETO	0 (0.0)	0.0–5.9	34 (25.0)	18.5–32.9
PAS	0 (0.0)	0.0–5.9	6 (4.4)	2.0–9.3
Pre-XDR	0 (0.0)	0.0–5.9	28 (20.6)	14.7–28.2
XDR	0 (0.0)	0.0–5.9	1 (0.7)	0.1–4.1

Excluding 2 isolates with disputed *rpoB* mutations and 1 streptomycin mono-resistant isolate.

*DR* drug resistance, *RR* rifampicin resistance, *MDR* multidrug resistance, *Pre-XDR* pre-extensively drug resistance, *XDR* extensively drug resistance, *CI* confidence interval, *RIF* rifampicin, *INH* isoniazid, *EMB* ethambutol, *PZA* pyrazinamide, *STR* streptomycin, *FQs* fluoroquinolones, *KM* kanamycin, *AMK* amikacin, *CM* capreomycin, *ETO* ethionamide, *PAS* para-aminosalicylic acid.

#### Genotypic drug resistance

**Figure 2:**
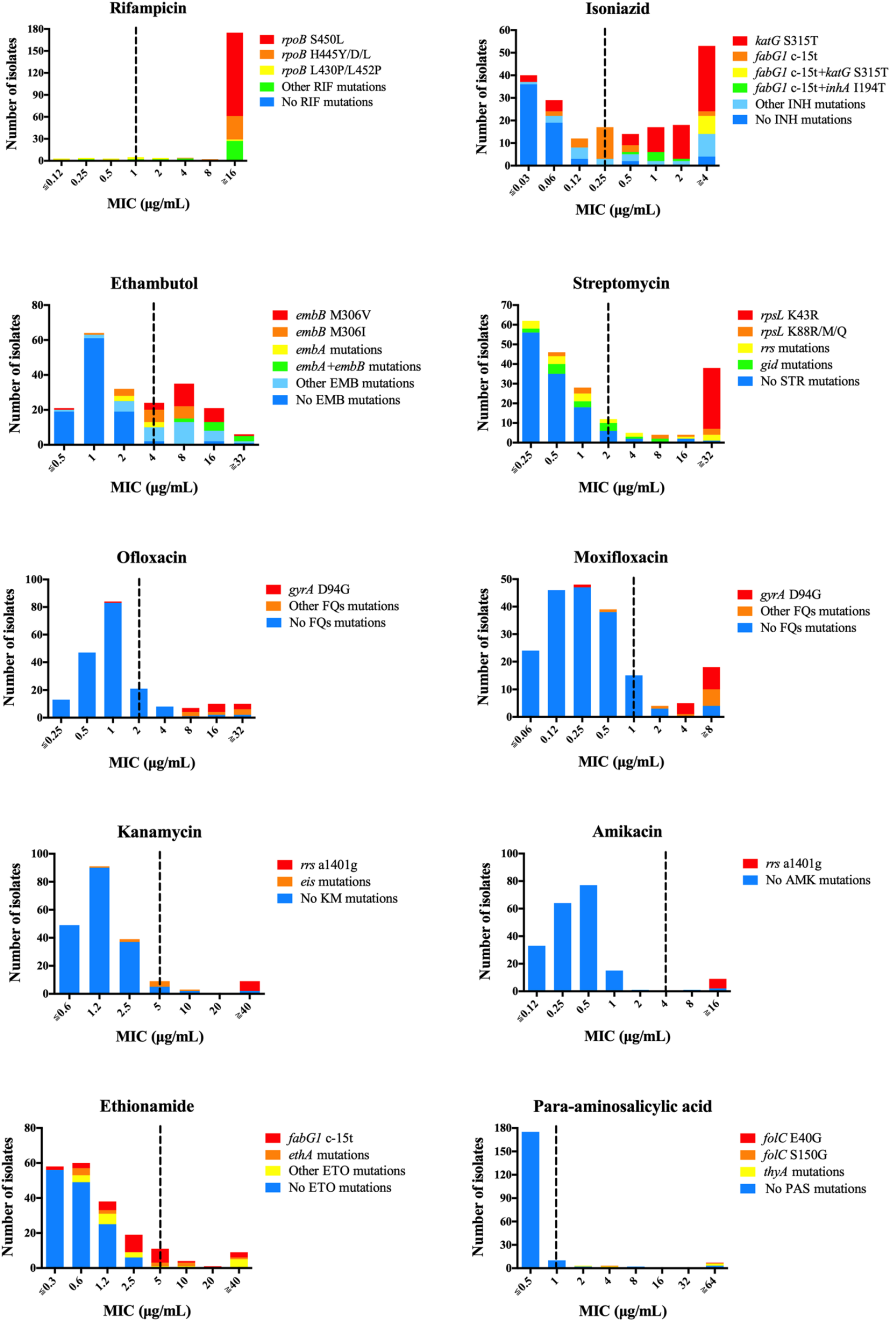
Distribution of drug resistance-associated mutations with corresponding MICs. Each stacked column represents a collection of isolates colored according to their genetic background. The x-axes show the MICs in μg/mL. The dashed lines indicates the critical concentrations used for MYCOTB plates.

Using the phenotypic DST results as a reference, the drug resistance-associated mutations and MIC distributions of the isolates were shown in Supplementary Table S2 and Fig. 2, respectively. The confidence level for grading mutations was based on the 2021 WHO catalog of *M. tuberculosis* mutations^18^. In addition, the candidate mutations identified by the TGS-TB database, including *fabG1* L203L, *rpoB* L430P, L452P, were classified as genotypically resistant according to the WHO mutations catalog.

#### Rifampicin resistance

Among the 197 phenotypically RIF-resistant isolates, 120 (60.9%) isolates had high-confidence *rpoB* S450L and 25 (12.7%) isolates with H445Y, which showed MICs ≥ 4 μg/mL. Four isolates concurrently exhibited *rpoB* S450L and putative compensatory *rpoC* mutations, I491T, G332R, F452S, and L527V, with MICs > 16 μg/mL. Furthermore, six isolates presented disputed mutations, *rpoB* L430P or L452P, with concurrent mutations exhibited MICs > 16 μg/mL. In contrast, 69.2% of isolates with the single disputed mutation, *rpoB* L430P or L452P, exhibited MICs ≤ 1 μg/mL.

#### Isoniazid resistance

Among the 136 phenotypically INH-resistant isolates, 77 (56.6%) isolates had high-confidence *katG* S315T and 44 (32.4%) isolates with low-confidence *fabG1* c-15t. We found that 60 (88.2%) isolates with single *katG* S315T showed MICs ≥ 0.5 μg/mL, while 22 (88.0%) isolates with single *fabG1* c-15t showed MICs ≤ 0.5 μg/mL. The combination of *katG* S315T and *fabG1* c-15t was associated with elevated MICs (≥ 4 μg/mL). In addition, six INH-resistant isolates with concurrent *fabG1* c-15t and *inhA* I194T mutations also presented MICs ≥ 0.5 μg/mL. Five INH-susceptible isolates with *katG* S315T, *katG* W191R, *fabG1* c-15t, or *ahpC* c-52t exhibited MICs ≤ 0.12 μg/mL. Furthermore, the novel mutations, *katG* D329Y, G370E and P375L, with MICs ≤ 0.5 μg/mL, were each found in three isolates.

#### Ethambutol resistance

Among the 77 phenotypically EMB-resistant isolates, 25 (32.5%) isolates had high-confidence *embB* M306V and 15 (19.5%) isolates with M306I. Of the 41 isolates with single *embB* M306V/I, 17 (41.5%) isolates presented MICs ≤ 4 μg/mL, and 7 (17.1%) of them exhibited an EMB-susceptible phenotype. All six isolates with single *embA* mutations also presented MICs ≤ 4 μg/mL, and four of them exhibited an EMB-susceptible phenotype. Notably, isolates concurrently harboring *embA* and *embB* mutations were associated with elevated MICs (≥ 8 μg/mL).

#### Pyrazinamide resistance

Among the 40 phenotypically PZA-resistant isolates, 39 isolates harbored 36 types of mutations scattered throughout the *pncA* gene and promoter; thus, high diversity of *pncA* mutations was observed without major hot spots.

#### Streptomycin resistance

Among the 60 STR-resistant isolates, 31 (51.7%) isolates had high-confidence *rpsL* K43R, 9 (15%) isolates with K88R, and 7 (11.7%) isolates with *rrs* a514c. All 31 isolates with *rpsL* K43R presented high MICs (≥ 32 μg/mL), while 9 isolates with *rpsL* K88R presented wide range of MICs (0.5 to > 32 μg/mL). Among 20 isolates with *rrs* mutations, 14 (70%) of them exhibited MICs ≤ 2 μg/mL. Besides, of 18 isolates with *gid* mutations, even though mutations in the *gid* gene were associated with STR resistance, 15 (83.3%) isolates exhibited MICs ≤ 2 μg/mL.

#### Fluoroquinolones resistance

Among the 22 FQs-resistant isolates, 14 (63.6%) isolates had high-confidence *gyrA* D94G. All 14 FQs-resistant isolates with *gyrA* D94G presented high MICs (≥ 4 μg/mL for ofloxacin (OFX) and ≥ 2 μg/mL for MFX). Other *gyrA* mutations were also associated with high MICs (≥ 4 μg/mL for OFX and ≥ 2 μg/mL for MFX). In addition, we identified one novel *gyrB* G522S mutation.

#### Second-line injectable drug resistance

Cross-resistance among injectable drugs was associated with the high-confidence mutation *rrs* a1401g, which was found in seven KM-resistant isolates (63.6%) with MICs > 40 μg/mL and seven AMK-resistant isolates (87.5%) with MICs > 16 μg/mL, respectively. Moreover, all six isolates with *eis* c-12t exhibited a KM-susceptible phenotype.

#### Ethionamide resistance

Among the 34 ETO-resistant isolates, 25 (73.5%) isolates had low-confidence *fabG1* c-15t, which was cross-resistant to INH. Of the 33 isolates with single *fabG1* c-15t, 28 (84.8%) isolates exhibited MICs ≤ 5 μg/mL, and 14 (42.4%) of them exhibited an ETO-susceptible phenotype. Besides, isolates with *fabG1* c-15t with concurrent *inhA* I194T (n = 3, 50.0%) or *ethR* A95T (n = 5, 100.0%) exhibited MICs ≤ 5 μg/mL. In addition, of 11 isolates with single *ethA* frameshift mutations, 8 (72.7%) of them exhibited MICs ≤ 5 μg/mL.

#### Para-aminosalicylic acid resistance

Among six PAS-resistant isolates, one isolate carried *folC* E40G with MIC > 64 μg/mL, and the other five isolates harbored novel mutations, *thyA* L38S, L218P, R235W, and Y251stop with MICs = 2 to ≥ 64 μg/mL. One isolate with the *folC* S150G mutation was phenotypically PAS-susceptible with an MIC = 4 μg/mL.

### Performance of WGS in drug resistance prediction

The performance of WGS for the prediction of drug resistance was shown in Table 3. The average concordance was 94.9%, ranging from 84.0% (ETO) to 99.5% (FQs, AMK and PAS). The overall sensitivity and specificity of WGS-based DST were 97.2% and 94.0%, respectively. The sensitivity of WGS to predict resistance to INH (96.3%), FQs (100.0%) and PAS (100.0%) were further improved by inclusion of novel mutations, *katG* D329Y, G370E, P375L, *gyrB* G522S, and *thyA* L38S, L218P, R235W, Y251stop (Table 3). Excluding SLIDs, the resistance predictive values of other tested drugs were higher than 95.0%. In addition, three isolates harboring *rpoB* L430P or L452P disputed mutations were phenotypically RIF susceptible, which resulted in low specificity.

**Table 3:** Performance of whole-genome sequencing in predicting drug-resistance.

Drugs	pDST resistance		pDST susceptible			Performance (Excluding novel mutations)			Performance (Including novel mutations)		
	gDST (No.)		gDST (No.)			Concordance (%)	Sensitivity (%)	Specificity (%)	Concordance (%)	Sensitivity (%)	Specificity (%)
	R^a^	U^b^	S^c^	R^a^	S^c^						
RIF	196	0	1	3	0	98.0	99.5	0.0	98.0	99.5	0.0
INH	128	3	5	5	59	93.5	94.1	92.2	95.0	96.3	92.2
EMB	74	0	3	22	101	87.5	96.1	82.1	87.5	96.1	82.1
PZA	39	0	1	2	158	98.5	97.5	98.8	98.5	97.5	98.8
STR	58	0	2	22	118	88.0	96.7	84.3	88.0	96.7	84.3
FQs	21	1	0	1	177	99.0	95.5	99.4	99.5	100.0	99.4
KM	9	0	2	6	183	96.0	81.8	96.8	96.0	81.8	96.8
AMK	7	0	1	0	192	99.5	87.5	100.0	99.5	87.5	100.0
CM	5	0	1	3	191	98.0	83.3	98.5	98.0	83.3	98.5
ETO	33	0	1	31	135	84.0	97.1	81.3	84.0	97.1	81.3
PAS	1	5	0	1	193	97.0	16.7	99.5	99.5	100.0	99.5
Overall	571	9	17	96	1507	94.5	95.6	94.0	94.9	97.2	94.0

*RIF* rifampicin, *INH* isoniazid, *EMB* ethambutol, *PZA* pyrazinamide, *STR* streptomycin, *FQs* fluoroquinolones, *KM* kanamycin, *AMK* amikacin, *CM* capreomycin, *ETO* ethionamide, *PAS* para-aminosalicylic acid, *pDST* phenotypic drug susceptibility testing, *gDST* genotypic drug susceptibility testing.

^a^R, detection of resistance-associated mutations.

^b^U, detection of novel mutations association with resistance unknown.

^c^S, detection of mutations known not to be associated with resistance (phylogenetic marker or synonymous mutation) or no mutation detect.

### Associations between lineages and drug-resistance

**Figure 3:**
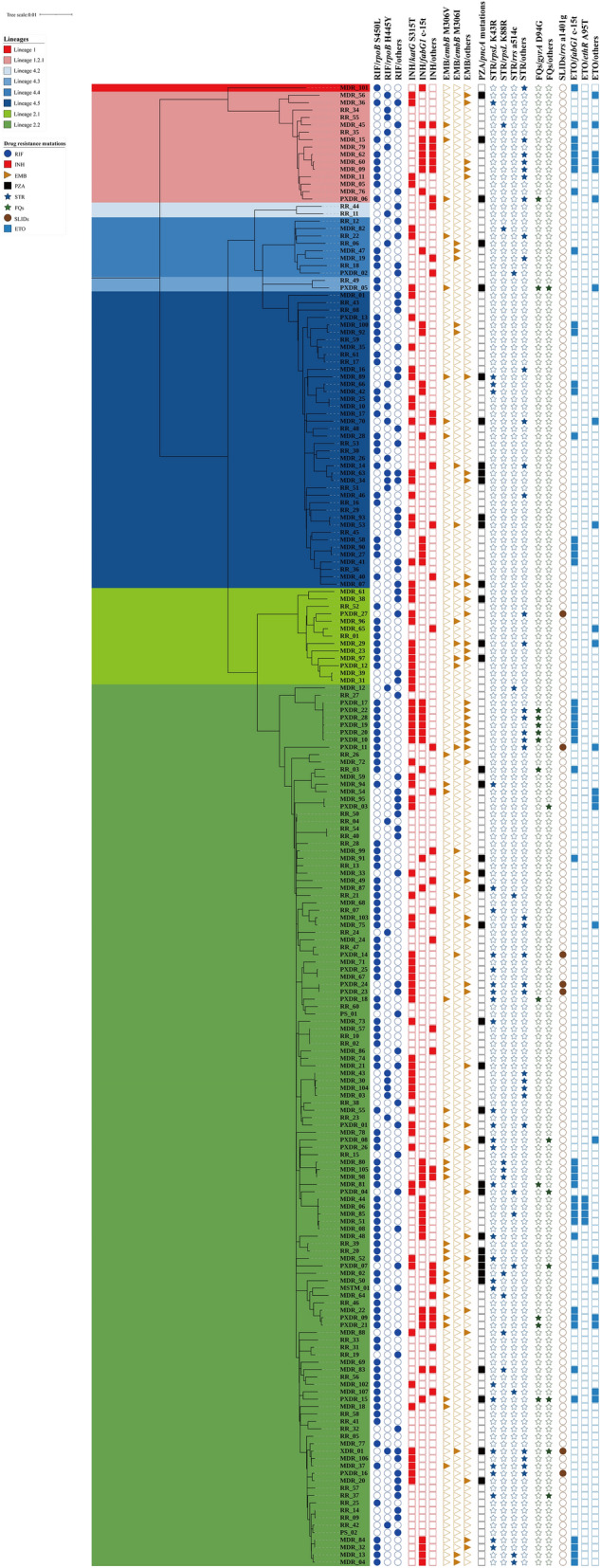
Maximum likelihood phylogenetic tree of the 200 DR-TB isolates from Taiwan. The tree was constructed based on 12,015 genome-wide SNPs. Lineages are represented by different colored blocks. Mutations are represented by filled (presence of mutation) or empty (absence of mutation) symbols. The figure was generated using iTOL v6 (https://itol.embl.de). The scale bar indicates the genetic distance proportional to the total number of SNPs. Abbreviations: RIF, rifampicin; INH, isoniazid; EMB, ethambutol; PZA, pyrazinamide; STR, streptomycin; FQs, fluoroquinolones; SLIDs, second-line injectable drugs; ETO, ethionamide; MDR, multidrug resistant; RR, rifampicin resistant; PXDR, pre-extensively drug resistant; XDR, extensively drug resistant; MSTM, mono-streptomycin resistant; PS, pansusceptible.

We constructed a maximum likelihood phylogenetic tree based on 12,015 SNP differences (Fig. 3). Lineage 2 isolates were significantly resistant to EMB and STR than lineage 1 and lineage 4 (*P* < 0.05) (Supplementary Table S3). Lineage 1 isolates were significantly resistant to ETO when compared to lineage 2 and lineage 4 (*P* < 0.05) (Supplementary Table S3). Furthermore, we identified lineage-specific variants, such as RIF *rpoB* S450L was predominant in lineage 2 (65.2%, *P* = 0.038); RIF *rpoB* H445Y was significantly associated with lineage 1 (31.3%, *P* = 0.034); INH *fabG1* c-15t was significantly associated with lineage 1 (50.0%, *P* = 0.011); STR *rpsL* K43R was significantly associated with lineage 2 (20.5%, *P* = 0.007) (Fig. 3, Table 4).

**Table 4:** Profiles of drug-resistant mutations in lineage 1, lineage 2 and lineage 4 isolates.

Drugs/gene mutations	Lineage 1 (N = 16)			Lineage 2 (N = 132)			Lineage 4 (N = 52)		
	No. (%)	OR (95% CI)	*P* value	No. (%)	OR (95% CI)	*P* value	No. (%)	OR (95% CI)	*P* value
RIF/*rpoB* S450L	9 (56.3)	0.85 (0.30–2.37)	0.752	86 (65.2)	1.87 (1.03–3.39)	**0.038**	25 (48.1)	0.52 (0.27–0.98)	**0.041**
RIF/*rpoB* H445Y	5 (31.3)	3.72 (1.17–11.83)	**0.034**	11 (8.3)	0.35 (0.15–0.82)	**0.013**	9 (17.3)	1.73 (0.71–4.19)	0.222
INH/*katG* S315T	4 (25.0)	0.48 (0.15–1.56)	0.290^a^	57 (43.2)	1.58 (0.86–2.94)	0.138	18 (34.6)	0.76 (0.39–1.46)	0.403
INH/*fabG1* c-15t	8 (50.0)	3.97 (1.40–11.29)	**0.011**	27 (20.5)	0.71 (0.36–1.42)	0.335	10 (19.2)	0.77 (0.35–1.69)	0.512
EMB/*embB* M306V	3 (18.8)	1.40 (0.37–5.26)	0.709^a^	21 (15.9)	1.42 (0.59–3.40)	0.431	5 (9.6)	0.55 (0.20–1.52)	0.245
EMB/*embB* M306I	0 (0.0)	NA	0.240^a^	11 (8.3)	0.68 (0.26–1.78)	0.435	8 (15.4)	2.26 (0.86–5.99)	0.104
PZA/*pncA* mutations	3 (18.8)	0.92 (0.25–3.38)	1.000^a^	27 (20.5)	1.09 (0.52–2.28)	0.823	10 (19.2)	0.94 (0.42–2.08)	0.862
STR/*rpsL* K43R	1 (6.3)	0.34 (0.04–2.69)	0.475^a^	27 (20.5)	4.11 (1.38–12.30)	**0.007**	3 (5.8)	0.26 (0.08–0.90)	**0.026**^**a**^
STR/*rpsL* K88R	1 (6.3)	1.47 (0.17–12.52)	1.000^a^	7 (5.3)	1.85 (0.37–9.15)	0.507^a^	1 (1.9)	0.34 (0.04–2.81)	0.451^a^
STR/*rrs* a514c	0 (0.0)	NA	0.620^a^	8 (6.1)	4.32 (0.53–35.30)	0.171^a^	1 (1.9)	0.34 (0.04–2.81)	0.451^a^
FQs/*gryA* D94G	1 (6.3)	0.81 (0.10–6.59)	1.000^a^	11 (8.3)	1.45 (0.45–4.75)	0.588^a^	3 (5.8)	0.69 (0.19–2.56)	0.764^a^
KM, AMK, CM/*rrs* a1401g	0 (0.0)	NA	1.000^a^	7 (5.3)	NA	0.098^a^	0 (0.0)	NA	0.194^a^
ETO/*fabG1* c-15t	8 (50.0)	3.97 (1.40–11.29)	**0.011**	27 (20.5)	0.71 (0.36–1.42)	0.335	10 (19.2)	0.77 (0.35–1.69)	0.512

Statistical significances are represented in bold.

*RIF* rifampicin, *INH* isoniazid, *EMB* ethambutol, *PZA* pyrazinamide, *STR* streptomycin, *FQs* fluoroquinolones, *KM* kanamycin, *AMK* amikacin, *CM* capreomycin, *ETO* ethionamide, *OR* odds ratio, *CI* confidence interval, *NA* not applicable due to a small no. of cases.

^a^Fisher’s exact probability test (two-tailed).

## Discussion

This is the first study to demonstrate that WGS/TGS-TB had excellent performance in drug resistance prediction and the genetic diversity identification of *M. tuberculosis* in Taiwan*.* The good concordance rates in the detection of drug resistance against RIF, INH, PZA and FQs ranged from 95.0 to 99.5%, which were comparable to 96.4–100.0% reported in a previous study^15^. Together with the MIC measurements, the novel mutations *katG* D329Y, G370E and *thyA* L38S might confer low resistance levels. The predominant lineage 2 East Asian (particularly Beijing 2.2.1) was associated with drug resistance, as previously suggested^19^. Besides, *rpoB* S450L and *rpsL* K43R were significantly prevalent in lineage 2. Collective information is useful for DR-TB diagnosis and care.

WGS and MICs data provided informative insights on MTBC drug resistance. Nevertheless, suboptimal agreement in predictions of resistance to EMB (87.5%), STR (88.0%) and ETO (84.0%) was mainly attributed to mutations conferring low resistance levels, clinical breakpoint artifacts in pDST, incomprehensive mutation catalogs, and unknown resistance mechanisms^20^. False-susceptible pDST results for EMB, STR, and ETO might occur because some mutations cause slight MIC increases close to the critical concentration (CC). Thus, the overlap between the MICs of mutant and wild-type isolates would result in misclassification based on pDST. These elevated MICs below current CCs may still be clinically meaningful due to a chance of higher drug resistance acquisition and risk treatment failure^20^.

The *fabG1* c-15t and *inhA* I194T mutation were associated with low-level INH resistance. In this study, six isolates with concurrent c-15t and I194T showed elevated MICs (≥ 0.5 μg/mL) and a previous study revealed that conferred high resistance levels and exhibited a synergistic effect on INH resistance^21^. In addition, *fabG1* c-15t was associated with cross-resistance between INH and the structurally related ETO. It is worth noting that two isolates with the *fabG1* L203L silent mutation were INH resistant (MDR_17 and MDR_40). This might occur through the upregulation of *fabG1* resulting from the creation of an alternative promoter for *fabG1* expression^22^. We found that isolates with frameshift and nonsense mutations in the *ethA* gene, encoding the EthA monooxygenase, might not be phenotypically resistance to ETO. The presence of other monooxygenases in *M. tuberculosis* might be able to compensate the inactivation of EthA^23^.

The low specificity and NPV were due to all three RIF-susceptible isolates carrying disputed *rpoB* L430P or L452P mutation, which exhibited low MICs (≤ 1 μg/mL). Previous studies have reported that isolates with disputed *rpoB* mutations, L430P, D435Y, H445C/L/N/S, and L452P, confer low levels of RIF resistance^24^. However, isolates harboring disputed mutations concurrent with R62C, Q67R/H, M434L, or D435G mutations presented high MIC values (≥ 16 μg/mL), as mentioned in our previous study^24^.

Isolates with *embB* mutations combined with EMB *embC-embA* intergenic region (IGR) mutations, such as *embA* c-11a, c-12t, and c-16t, could show increased MIC values. Mutations in the *embC-embA* IGR might enhance the binding of EmbR to the promoter region of *embAB* and increase the transcription of *embAB*, thus contributing to EMB resistance^25^. Mutations in *embB* M306V/I and G406D/S were found in both EMB-resistant and EMB-susceptible isolates. Previous studies reported that *embB* M306V/I mutations cause slight MIC increases close to the CC ^26^. The inconsistency of EMB between WGS and pDST may also be due to inappropriate CCs and poor repeatability of pDST^26^. In addition, the *embABC* operon is involved in the decaprenylphosphoryl-β-D-arabinose (DPA) biosynthetic and utilization pathway, which might alter cell wall permeability and cause variability in EMB MICs^27^. This implies that the *embB*306 mutation results in varying degrees of EMB resistance but does not cause high-level EMB resistance on its own^27^.

Mutations in the *pncA* gene leading to a reduction in pyrazinamidase (PZase) activity are the main mechanism of PZA resistance^28^. We found a high diversity of *pncA* gene mutations without major hot spots in the PZA-resistant isolates, consistently with previous studies^29,30^. Although the reason for this diversity is still unclear, it might be due to adaptive mutagenesis or deficiency in DNA mismatch repair mechanisms^31^. Mutations in the *gyrA* or *gyrB* genes are associated with FQs resistance^28^. In particular, isolates with *gyrA* D94G show high MICs. Our study revealed that *gyrA* D94G was the predominant mutation associated with high MIC values for OFX and MFX (Supplementary Table S2, Fig. 2) as previously reported^32^.

Mutations in the *rrs* gene, encodes the 16S rRNA, confer moderate levels of STR resistance^33^. Whereas, mutations in the *gid* gene, encodes a 7-methylguanosine methyltransferase, reduce 16S rRNA methylation, thereby interfering with STR binding and consequently conferring low levels of STR resistance^34^. Besides, *eis* promoter mutations, g-10a and c-14t, accounted for 33% of KM resistance^35^. The *eis* c-14t mutation conferred a higher level of KM resistance than the g-10a, g-37t, and c-12t mutations^36^. Nevertheless, no *eis* c-14t mutants were identified in this study.

Suboptimal prediction of resistance to KM (81.8%), AMK (87.5%), and CM (83.3%) might be a few resistant isolates analyzed, the presence of additional resistance mutations in genes not assessed, or to unknown resistance mechanisms. The mechanisms of drug resistance have yet to be fully elucidated. The strain genetic background, clonal interference, epistatic interactions, efflux pump mutations, target modification and mimicry could contribute to various levels of drug resistance^37^. Rv1258c encodes the homologous Tap protein in *M. tuberculosis*, which is regulated by transcriptional activator WhiB7^38^. An increase in *whiB7* expression, resulting from mutations located in the 5’ untranslated region, leads to upregulation of *eis* and *tap*, conferring low-level resistance to aminoglycosides^39^.

Lineage 2 and lineage 4 M*. tuberculosis* isolates were predominant in Taiwan (Fig. 1). Sublineage 2.2 and sublineage 4.5 were predominant in Taiwan as well as in East Asia^40^. In addition, lineage 1 isolates, particularly sublineage 1.2.1, were prevalent in south and southeast Asia^41^. Notably, we found geographic disparities in sublineage 1.2.1 isolates mainly found in southern Taiwan, where the majority of migrants live, and none were identified in eastern Taiwan. Phylogenetic analysis showed that drug resistance mutations, RIF *rpoB* H445Y, was associated with lineage 1, as observed in a previous study^42^; whereas, RIF *rpoB* S450L and STR *rpsL* K43R, were associated with lineage 2, as observed in previous studies^19,43^. Higher mutation rates of lineage 2 isolates might account for increased adaptation abilities and drug resistance rates^44^.

Several software tools were available for predicting the drug resistance of *M. tuberculosis*, including PhyResSE, MyKrobe Predictor, KvarQ, TB profiler and TGS-TB^6^. However, performance of drug resistance prediction varies between the different tools and anti-TB drugs tested^6,45^. A previous study revealed that the sensitivity of PZA resistance prediction was higher using TGS-TB (87.0%) than that using TB profiler (< 65.0%)^45^. The major difference between TGS-TB and TB profiler in PZA resistance prediction was due to the inclusion of insertions and deletions associated with PZA resistance^46^. Additionally, the performances of PhyResSE, MyKrobe Predictor and KvarQ were unsatisfactory for predicting resistance to PZA and EMB^6^. Notably, TGS-TB was much more user friendly as compared to other tools for WGS data analysis and could process online batch analysis for multiple samples.

The study has some limitations. Firstly, due to the low resistant rates (< 10%) of certain study drugs, KM, AMK, CM, and PAS and few fully susceptible isolates were analyzed. As results, biases on performance might occur. Secondly, there was no MIC testing for PZA and CM to compare WGS with the level of phenotypic resistance. Thirdly, lineages may affect the prediction of drug resistance by WGS and was not take into account. Lastly, besides the genetically-encoded determinants, changes in transcription or translation may also mediate antibiotic tolerance and persistence state, which also impact the efficacy of antibiotics in vivo^47^.

Phenotypic DST for the prediction of TB drug resistance has limitations, hampering timely personalized precision therapy and comprehensive surveillance. To strengthen and revolutionize the DR-TB control program, WGS provides a solution for genetic drug resistance prediction and surveillance of existing, new, and repurposed TB drugs with satisfactory accuracy. This pilot study demonstrated the feasibility of the application of WGS for TB control programs in Taiwan. Notably, a diagnostic policy to streamline and integrate WGS into our routine TB laboratory services for analyzing *M. tuberculosis* isolated from all new RR/MDR cases has been established since 2019. We expect to expand the services to DR-TB cases with other drug-resistant patterns if the resource is available. In line with some high-income countries, this study reassures that WGS is a valuable tool to inform clinical and public health actions. Our results could serve as a guide to facilitate the uptake of new technology in the TB control program.

## Supplementary Information

Supplementary Information.

## Data Availability

Sequencing reads have been submitted to the National Center for Biotechnology Information (NCBI) Sequence Read Archive (SRA) under BioProject ID PRJNA879962.
