# Supplementary material for: Whole-genome sequencing-based analyses of drug-resistant Mycobacterium tuberculosis from Taiwan
Source: Sci Rep. 2023 Feb 13;13:2540. doi: 10.1038/s41598-023-29652-3 (PMC9925824; doi:10.1038/s41598-023-29652-3)
Supplement: Supplementary file 1 — Supplementary Information. [file 41598_2023_29652_MOESM1_ESM.docx]

**Whole-genome sequencing-based analyses of drug-resistant *Mycobacterium tuberculosis* from Taiwan**

**Supplementary Tables**

| **Table S1. Phenotypic drug resistance patterns of 200 *Mycobacterium tuberculosis* isolates.** | | |
| --- | --- | --- |
| Drug-resistant profiles |  | No. of isolates (%) |
| Pansusceptible^a^ |  | 2 (1.0) |
| Mono-STR resistance |  | 1 (0.5) |
| RR |  | 61 (30.5) |
| RIF only |  | 55 (27.5) |
| RIF+EMB |  | 1 (0.5) |
| RIF+PZA |  | 1 (0.5) |
| RIF+STR |  | 1 (0.5) |
| RIF+PZA+FQs |  | 1 (0.5) |
| RIF+STR+FQs |  | 1 (0.5) |
| RIF+FQs+SLIDs |  | 1 (0.5) |
| MDR |  | 107 (53.5) |
| RIF+INH |  | 33 (16.5) |
| RIF+INH+EMB |  | 12 (6.0) |
| RIF+INH+PZA |  | 2 (1.0) |
| RIF+INH+STR |  | 7 (3.5) |
| RIF+INH+ETO |  | 7 (3.5) |
| RIF+INH+EMB+PZA |  | 9 (4.5) |
| RIF+INH+EMB+STR |  | 6 (3.0) |
| RIF+INH+EMB+ETO |  | 4 (2.0) |
| RIF+INH+PZA+STR |  | 1 (0.5) |
| RIF+INH+PZA+ETO |  | 1 (0.5) |
| RIF+INH+STR+ETO |  | 3 (1.5) |
| RIF+INH+EMB+PZA+STR |  | 12 (6.0) |
| RIF+INH+EMB+PZA+ETO |  | 1 (0.5) |
| RIF+INH+EMB+STR+ETO |  | 5 (2.5) |
| RIF+INH+PZA+STR+ETO |  | 1 (0.5) |
| RIF+INH+EMB+PZA+STR+ETO |  | 2 (1.0) |
| RIF+INH+EMB+STR+ETO+PAS |  | 1 (0.5) |
| Pre-XDR |  | 28 (14.0) |
| RIF+INH+FQs |  | 1 (0.5) |
| RIF+INH+EMB+FQs |  | 2 (1.0) |
| RIF+INH+EMB+SLIDs |  | 2 (1.0) |
| RIF+INH+STR+SLIDs |  | 3 (1.5) |
| RIF+INH+ETO+FQs |  | 1 (0.5) |
| RIF+INH+EMB+STR+FQs |  | 1 (0.5) |
| RIF+INH+EMB+STR+SLIDs |  | 5 (2.5) |
| RIF+INH+EMB+PZA+STR+FQs |  | 1 (0.5) |
| RIF+INH+EMB+PZA+ETO+FQs |  | 1 (0.5) |
| RIF+INH+EMB+PZA+PAS+FQs |  | 2 (1.0) |
| RIF+INH+EMB+STR+ETO+FQs |  | 3 (1.5) |
| RIF+INH+EMB+STR+PAS+FQs |  | 1 (0.5) |
| RIF+INH+EMB+ETO+PAS+FQs |  | 1 (0.5) |
| RIF+INH+EMB+PZA+STR+ETO+FQs |  | 3 (1.5) |
| RIF+INH+EMB+PZA+STR+PAS+FQs |  | 1 (0.5) |
| XDR |  | 1 (0.5) |
| RIF+INH+EMB+PZA+STR+FQs+SLIDs |  | 1. (0.5) |
| ^a^ Isolate with disputed *rpoB* mutations.  Abbreviations: RIF, rifampicin; INH, isoniazid; EMB, ethambutol; PZA, pyrazinamide; STR, streptomycin; FQs, fluoroquinolones; KM, kanamycin; AMK, amikacin; CM, capreomycin; ETO, ethionamide; PAS, para-aminosalicylic acid; RR, rifampicin resistance; MDR, multidrug resistance; Pre-XDR, pre-extensively drug resistance; XDR, extensively drug resistance. | | |

| \| **Table S2. Catalog of drug resistance-associated mutations with MIC distribution of 200 *Mycobacterium tuberculosis* isolates.** \| \| \| \| \| \| \| \| \| \| \| \| \| \| \| --- \| --- \| --- \| --- \| --- \| --- \| --- \| --- \| --- \| --- \| --- \| --- \| --- \| --- \| \| Drug \| pDST  (No. of isolates, %) \| Mutations \| MIC (μg/ml) distribution \| \| \| \| \| \| \| \| \| No. of isolates \| % \| \| \| RIF \|  \|  \| ≤0.12 \| 0.25 \| 0.5 \| 1 \| 2 \| 4 \| 8 \| 16 \| >16 \|  \|  \| \| \|  \| Resistant  (197, 98.5%) \| *rpoB* S450L \|  \|  \|  \|  \|  \|  \|  \| 9 \| 105 \| 114 \| 57.9 \| \| \| *rpoB* S450L, *rpoB* I480T \|  \|  \|  \|  \|  \|  \|  \|  \| 1 \| 1 \| 0.5 \| \| \| *rpoB* S450L, *rpoB* I480V \|  \|  \|  \|  \|  \|  \|  \|  \| 1 \| 1 \| 0.5 \| \| \| *rpoB* S450L, *rpoC* G332R \|  \|  \|  \|  \|  \|  \|  \|  \| 1 \| 1 \| 0.5 \| \| \| *rpoB* S450L, *rpoC* F452S \|  \|  \|  \|  \|  \|  \|  \|  \| 1 \| 1 \| 0.5 \| \| \| *rpoB* S450L, *rpoC* I491T \|  \|  \|  \|  \|  \|  \|  \|  \| 1 \| 1 \| 0.5 \| \| \| *rpoB* S450L, *rpoC* L527V \|  \|  \|  \|  \|  \|  \|  \|  \| 1 \| 1 \| 0.5 \| \| \| *rpoB* S450W \|  \|  \|  \|  \|  \|  \|  \|  \| 2 \| 2 \| 1.0 \| \| \| *rpoB* H445Y \|  \|  \|  \|  \|  \|  \|  \| 1 \| 20 \| 21 \| 10.7 \| \| \| *rpoB* H445Y, *rpoB* L430P \|  \|  \|  \|  \|  \|  \|  \|  \| 1 \| 1 \| 0.5 \| \| \| *rpoB* H445Y, *rpoB* T427P \|  \|  \|  \|  \|  \|  \|  \|  \| 2 \| 2 \| 1.0 \| \| \| *rpoB* H445Y, *rpoB* E460G \|  \|  \|  \|  \|  \|  \|  \|  \| 1 \| 1 \| 0.5 \| \| \| *rpoB* H445C \|  \|  \|  \|  \| 1 \|  \|  \|  \|  \| 1 \| 0.5 \| \| \| *rpoB* H445D \|  \|  \|  \|  \|  \|  \|  \|  \| 9 \| 9 \| 4.6 \| \| \| *rpoB* H445L \|  \|  \|  \|  \|  \| 1 \| 1 \|  \| 2 \| 4 \| 2.0 \| \| \| *rpoB* H445N, *rpoB* D435E \|  \|  \|  \|  \|  \|  \| 1 \|  \|  \| 1 \| 0.5 \| \| \| *rpoB* H445R \|  \|  \|  \|  \|  \|  \|  \|  \| 1 \| 1 \| 0.5 \| \| \| *rpoB* H445R, *rpoB* E460G \|  \|  \|  \|  \|  \|  \|  \|  \| 1 \| 1 \| 0.5 \| \| \| *rpoB* H445S \|  \|  \|  \|  \| 1 \|  \|  \|  \|  \| 1 \| 0.5 \| \| \| *rpoB* L430P, *rpoB* V170F \|  \|  \|  \|  \|  \|  \|  \|  \| 1 \| 1 \| 0.5 \| \| \| *rpoB* L430P, *rpoB* M434L \|  \|  \|  \|  \|  \|  \|  \|  \| 1 \| 1 \| 0.5 \| \| \| *rpoB* L430P, *rpoB* D435G \|  \|  \|  \|  \|  \|  \|  \|  \| 1 \| 1 \| 0.5 \| \| \| *rpoB* L452P \| 1 \| 1 \| 2 \| 2 \| 2 \|  \|  \|  \| 2 \| 10 \| 5.1 \| \| \| *rpoB* L452P, *rpoB* D435Y \|  \|  \|  \|  \|  \|  \|  \|  \| 1 \| 1 \| 0.5 \| \| \| *rpoB* L452P, *rpoB* D435Y, *rpoB* N437D \|  \|  \|  \|  \|  \|  \|  \|  \| 1 \| 1 \| 0.5 \| \| \| *rpoB* D435V \|  \| 1 \| 1 \|  \|  \| 2 \|  \|  \| 2 \| 6 \| 3.0 \| \| \| *rpoB* S441L \|  \|  \|  \|  \|  \|  \|  \|  \| 2 \| 2 \| 1.0 \| \| \| *rpoB* S441W, *rpoB* D435G \|  \| 1 \|  \|  \|  \|  \|  \|  \|  \| 1 \| 0.5 \| \| \| *rpoB* G442V \| 1 \|  \|  \|  \|  \|  \|  \|  \|  \| 1 \| 0.5 \| \| \| *rpoB* I491F \|  \|  \|  \|  \|  \| 1 \|  \|  \|  \| 1 \| 0.5 \| \| \| *rpoB* V170F \|  \|  \|  \|  \|  \|  \|  \|  \| 2 \| 2 \| 1.0 \| \| \| *rpoB* c.1298_1301insCTT \|  \|  \|  \|  \|  \|  \|  \| 1 \|  \| 1 \| 0.5 \| \| \| *rpoB* c.1306_1308delCAG \|  \|  \|  \| 2 \|  \|  \|  \|  \|  \| 2 \| 1.0 \| \| \| WT \|  \|  \|  \|  \|  \|  \|  \|  \| 1 \| 1 \| 0.5 \| \| \| Susceptible  (3, 1.5%) \| *rpoB* L430P \| 1 \|  \|  \|  \|  \|  \|  \|  \|  \| 1 \| 33.3 \| \| \| *rpoB* L452P \|  \| 1 \|  \| 1 \|  \|  \|  \|  \|  \| 2 \| 66.7 \| \| \| INH \|  \|  \| ≤0.03 \| 0.06 \| 0.12 \| 0.25 \| 0.5 \| 1 \| 2 \| 4 \| >4 \|  \|  \| \| \|  \| Resistant  (136, 68.0%) \| *katG* S315T \| 2 \| 4 \|  \|  \| 5 \| 11 \| 15 \| 20 \| 9 \| 66 \| 48.5 \| \| \| *katG* S315T, *inhA* S94A \|  \|  \|  \|  \|  \|  \|  \|  \| 1 \| 1 \| 0.7 \| \| \| *katG* S315T, *fabG1* t-8a \|  \|  \|  \|  \|  \|  \|  \| 1 \|  \| 1 \| 0.7 \| \| \| *katG* S315T *fabG1* c-15t \|  \|  \|  \|  \|  \|  \|  \| 1 \| 7 \| 8 \| 5.9 \| \| \| *katG* S315T, *fabG1* g-17t \|  \|  \|  \|  \|  \|  \|  \|  \| 1 \| 1 \| 0.7 \| \| \| *katG* Q127P \|  \|  \|  \|  \|  \|  \|  \|  \| 1 \| 1 \| 0.7 \| \| \| *katG* D329Y \|  \|  \| 1 \|  \|  \|  \|  \|  \|  \| 1 \| 0.7 \| \| \| *katG* G370E \|  \|  \| 1 \|  \|  \|  \|  \|  \|  \| 1 \| 0.7 \| \| \| *katG* P375L \|  \|  \|  \|  \| 1 \|  \|  \|  \|  \| 1 \| 0.7 \| \| \| *katG* deletion \|  \|  \|  \|  \|  \|  \|  \|  \| 1 \| 1 \| 0.7 \| \| \| *fabG1* c-15t \|  \| 2 \| 3 \| 14 \| 3 \|  \|  \| 2 \|  \| 24 \| 17.6 \| \| \| *fabG1* c-15t, *inhA* I194T \|  \|  \|  \|  \| 1 \| 4 \| 1 \|  \|  \| 6 \| 4.4 \| \| \| *fabG1* c-15t, *katG* A106V \|  \|  \|  \|  \|  \| 1 \|  \|  \|  \| 1 \| 0.7 \| \| \| *fabG1* c-15t, *katG* Q127P \|  \| 1 \|  \|  \|  \|  \| 1 \|  \|  \| 2 \| 1.5 \| \| \| *fabG1* c-15t, *katG* S140N, *katG* S315N \|  \|  \|  \|  \|  \|  \| 1 \|  \|  \| 1 \| 0.7 \| \| \| *fabG1* c-15t, *katG* c.1378_1381insGGA \|  \|  \|  \|  \| 1 \| 1 \|  \|  \|  \| 2 \| 1.5 \| \| \| *fabG1* t-8c \|  \|  \| 2 \| 1 \|  \|  \|  \|  \|  \| 3 \| 2.2 \| \| \| *fabG1* t-8c, *katG* G593D \|  \|  \| 1 \|  \|  \|  \|  \|  \|  \| 1 \| 0.7 \| \| \| *fabG1* L203L \|  \|  \|  \| 2 \|  \|  \|  \|  \|  \| 2 \| 1.5 \| \| \| *ahpC* g-48a, *katG* c.955_957insTG \|  \|  \|  \|  \|  \|  \|  \|  \| 1 \| 1 \| 0.7 \| \| \| *ahpC* g-48a, *katG* deletion \|  \|  \|  \|  \|  \|  \|  \|  \| 1 \| 1 \| 0.7 \| \| \| *ahpC* c-52t \|  \|  \|  \|  \|  \|  \|  \|  \| 2 \| 2 \| 1.5 \| \| \| *ahpC* c-54t \|  \|  \|  \|  \|  \|  \|  \|  \| 1 \| 1 \| 0.7 \| \| \| *ahpC* c-54t, *katG*_promoter a-10c \|  \|  \|  \|  \| 1 \|  \|  \|  \|  \| 1 \| 0.7 \| \| \| *ahpC* g-74a \|  \| 1 \|  \|  \|  \|  \|  \|  \|  \| 1 \| 0.7 \| \| \| WT \|  \| 1 \|  \|  \|  \|  \|  \| 1 \| 3 \| 5 \| 3.7 \| \| \| Susceptible  (64, 32.0%) \| *katG* W191R \|  \| 1 \|  \|  \|  \|  \|  \|  \|  \| 1 \| 1.6 \| \| \| *katG* S315T \| 1 \| 1 \|  \|  \|  \|  \|  \|  \|  \| 2 \| 3.1 \| \| \| *fabG1* c-15t \|  \|  \| 1 \|  \|  \|  \|  \|  \|  \| 1 \| 1.6 \| \| \| *ahpC* c-52t \| 1 \|  \|  \|  \|  \|  \|  \|  \|  \| 1 \| 1.6 \| \| \| WT \| 36 \| 18 \| 3 \|  \| 2 \|  \|  \|  \|  \| 59 \| 92.2 \| \| \| EMB \|  \|  \| ≤0.5 \| 1 \| 2 \| 4 \| 8 \| 16 \| 32 \| >32 \|  \|  \|  \| \| \|  \| Resistant  (77, 38.5%) \| *embB* M306V \|  \|  \|  \| 4 \| 11 \| 7 \| 1 \|  \|  \| 23 \| 29.9 \| \| \| *embB* M306V, *embB* D1024N \|  \|  \|  \|  \|  \|  \| 1 \|  \|  \| 1 \| 1.3 \| \| \| *embB* M306V, *embA* c-16t \|  \|  \|  \|  \|  \| 1 \|  \|  \|  \| 1 \| 1.3 \| \| \| *embB* M306I \|  \| 1 \| 2 \| 6 \| 2 \|  \|  \|  \|  \| 11 \| 14.3 \| \| \| *embB* M306I, *embB* G406D \|  \|  \|  \|  \|  \| 2 \|  \|  \|  \| 2 \| 2.6 \| \| \| *embB* M306I, *embA* c-12t \|  \|  \|  \|  \|  \|  \|  \| 1 \|  \| 1 \| 1.3 \| \| \| *embB* M306I, *embA* c-16t \|  \|  \|  \|  \| 1 \|  \|  \|  \|  \| 1 \| 1.3 \| \| \| *embB* M306L \|  \|  \| 1 \|  \|  \|  \|  \|  \|  \| 1 \| 1.3 \| \| \| *embB* G406A \|  \|  \|  \|  \|  \| 1 \|  \|  \|  \| 1 \| 1.3 \| \| \| *embB* G406A, *embA* c-15g \|  \|  \|  \|  \|  \|  \| 1 \|  \|  \| 1 \| 1.3 \| \| \| *embB* G406D \|  \| 1 \|  \|  \|  \| 1 \|  \|  \|  \| 2 \| 2.6 \| \| \| *embB* G406S \|  \|  \|  \| 1 \|  \|  \|  \|  \|  \| 1 \| 1.3 \| \| \| *embB* G406S, *embA* c-12t \|  \|  \|  \|  \| 1 \|  \|  \|  \|  \| 1 \| 1.3 \| \| \| *embB* Y319C \|  \|  \|  \|  \| 1 \|  \|  \|  \|  \| 1 \| 1.3 \| \| \| *embB* Y319S, *embA* c-16t \|  \|  \|  \|  \|  \| 1 \|  \|  \|  \| 1 \| 1.3 \| \| \| *embB* D328Y \|  \|  \| 2 \|  \|  \| 1 \|  \|  \|  \| 3 \| 3.9 \| \| \| *embB* D328Y, *embB* Y334H \|  \|  \|  \|  \|  \| 1 \|  \|  \|  \| 1 \| 1.3 \| \| \| *embB* Q497K \|  \|  \|  \| 2 \|  \|  \|  \|  \|  \| 2 \| 2.6 \| \| \| *embB* Q497R \|  \|  \| 1 \|  \| 6 \|  \|  \|  \|  \| 7 \| 9.1 \| \| \| *embB* D1024N \| 1 \|  \|  \|  \| 3 \| 1 \|  \|  \|  \| 5 \| 6.5 \| \| \| *embC* V757A \|  \| 1 \|  \|  \|  \|  \|  \|  \|  \| 1 \| 1.3 \| \| \| *embA* c-11a \|  \|  \|  \| 1 \|  \|  \|  \|  \|  \| 1 \| 1.3 \| \| \| *embA* c-12t \|  \|  \|  \| 1 \|  \|  \|  \|  \|  \| 1 \| 1.3 \| \| \| *embA* g-43c, *embB* G406A \|  \|  \|  \|  \|  \| 1 \|  \|  \|  \| 1 \| 1.3 \| \| \| *embA* g-43c, *embB* N399T \|  \|  \|  \|  \|  \| 2 \|  \|  \|  \| 2 \| 2.6 \| \| \| *embA* g-43c, *embB* I563L \|  \|  \|  \|  \|  \|  \| 1 \|  \|  \| 1 \| 1.3 \| \| \| WT \|  \| 1 \|  \|  \|  \| 2 \|  \|  \|  \| 3 \| 3.9 \| \| \| Susceptible  (123, 61.5%) \| *embB* M306V \| 1 \|  \|  \|  \| 2 \| 1 \|  \|  \|  \| 4 \| 3.3 \| \| \| *embB* M306I \|  \|  \| 2 \| 1 \|  \|  \|  \|  \|  \| 3 \| 2.4 \| \| \| *embB* M306I, *embB* G406A \|  \|  \| 1 \|  \|  \|  \|  \|  \|  \| 1 \| 0.8 \| \| \| *embB* G406A \|  \|  \|  \| 1 \|  \|  \|  \|  \|  \| 1 \| 0.8 \| \| \| *embB* G406D \|  \|  \|  \|  \| 2 \|  \|  \|  \|  \| 2 \| 1.6 \| \| \| *embB* G406S \|  \|  \|  \| 1 \|  \|  \|  \|  \|  \| 1 \| 0.8 \| \| \| *embB* Y319C \|  \| 1 \|  \|  \|  \|  \|  \|  \|  \| 1 \| 0.8 \| \| \| *embB* D328G \|  \|  \|  \| 2 \|  \|  \|  \|  \|  \| 2 \| 1.6 \| \| \| *embB* W332R \|  \|  \| 1 \|  \|  \|  \|  \|  \|  \| 1 \| 0.8 \| \| \| *embB* L402V \|  \|  \|  \| 1 \|  \|  \|  \|  \|  \| 1 \| 0.8 \| \| \| *embB* D1024N \|  \|  \|  \|  \| 1 \|  \|  \|  \|  \| 1 \| 0.8 \| \| \| *embA* c-11a \|  \|  \|  \| 1 \|  \|  \|  \|  \|  \| 1 \| 0.8 \| \| \| *embA* c-12t \|  \|  \| 2 \|  \|  \|  \|  \|  \|  \| 2 \| 1.6 \| \| \| *embA* c-16t \|  \|  \| 1 \|  \|  \|  \|  \|  \|  \| 1 \| 0.8 \| \| \| WT \| 19 \| 60 \| 19 \| 2 \|  \|  \|  \| 1 \|  \| 101 \| 82.1 \| \| \| PZA \|  \|  \|  \|  \|  \|  \|  \|  \|  \|  \|  \|  \|  \| \| \|  \| Resistant  (40, 20.0%) \| *pncA*_promoter a-11g \|  \|  \|  \|  \|  \|  \|  \|  \|  \| 1 \| 2.5 \| \| \| *pncA* C14R \|  \|  \|  \|  \|  \|  \|  \|  \|  \| 2 \| 5.0 \| \| \| *pncA* Y103stop \|  \|  \|  \|  \|  \|  \|  \|  \|  \| 2 \| 5.0 \| \| \| *pncA* M1T \|  \|  \|  \|  \|  \|  \|  \|  \|  \| 1 \| 2.5 \| \| \| *pncA* V7G \|  \|  \|  \|  \|  \|  \|  \|  \|  \| 1 \| 2.5 \| \| \| *pncA* D8G \|  \|  \|  \|  \|  \|  \|  \|  \|  \| 1 \| 2.5 \| \| \| *pncA* D12N \|  \|  \|  \|  \|  \|  \|  \|  \|  \| 1 \| 2.5 \| \| \| *pncA* L27P \|  \|  \|  \|  \|  \|  \|  \|  \|  \| 1 \| 2.5 \| \| \| *pncA* D49A \|  \|  \|  \|  \|  \|  \|  \|  \|  \| 1 \| 2.5 \| \| \| *pncA* H51P \|  \|  \|  \|  \|  \|  \|  \|  \|  \| 1 \| 2.5 \| \| \| *pncA* H57Q \|  \|  \|  \|  \|  \|  \|  \|  \|  \| 1 \| 2.5 \| \| \| *pncA* H57R \|  \|  \|  \|  \|  \|  \|  \|  \|  \| 1 \| 2.5 \| \| \| *pncA* D63A \|  \|  \|  \|  \|  \|  \|  \|  \|  \| 1 \| 2.5 \| \| \| *pncA* S67P \|  \|  \|  \|  \|  \|  \|  \|  \|  \| 1 \| 2.5 \| \| \| *pncA* W68R \|  \|  \|  \|  \|  \|  \|  \|  \|  \| 1 \| 2.5 \| \| \| *pncA* C72R \|  \|  \|  \|  \|  \|  \|  \|  \|  \| 1 \| 2.5 \| \| \| *pncA* G78V \|  \|  \|  \|  \|  \|  \|  \|  \|  \| 1 \| 2.5 \| \| \| *pncA* G132S \|  \|  \|  \|  \|  \|  \|  \|  \|  \| 1 \| 2.5 \| \| \| *pncA* V139A \|  \|  \|  \|  \|  \|  \|  \|  \|  \| 1 \| 2.5 \| \| \| *pncA* V155G \|  \|  \|  \|  \|  \|  \|  \|  \|  \| 1 \| 2.5 \| \| \| *pncA* V155M \|  \|  \|  \|  \|  \|  \|  \|  \|  \| 1 \| 2.5 \| \| \| *pncA* V157G \|  \|  \|  \|  \|  \|  \|  \|  \|  \| 1 \| 2.5 \| \| \| *pncA* G162D \|  \|  \|  \|  \|  \|  \|  \|  \|  \| 1 \| 2.5 \| \| \| *pncA* L172P \|  \|  \|  \|  \|  \|  \|  \|  \|  \| 1 \| 2.5 \| \| \| *pncA* M175V \|  \|  \|  \|  \|  \|  \|  \|  \|  \| 1 \| 2.5 \| \| \| *pncA* stop187W \|  \|  \|  \|  \|  \|  \|  \|  \|  \| 1 \| 2.5 \| \| \| *pncA* c.74_78delCCGCG \|  \|  \|  \|  \|  \|  \|  \|  \|  \| 1 \| 2.5 \| \| \| *pncA* c.164_170delGTGACCA \|  \|  \|  \|  \|  \|  \|  \|  \|  \| 1 \| 2.5 \| \| \| *pncA* c.170_178delACTTCTCCG \|  \|  \|  \|  \|  \|  \|  \|  \|  \| 1 \| 2.5 \| \| \| *pncA* c.387_395delTGTGGTCGG \|  \|  \|  \|  \|  \|  \|  \|  \|  \| 1 \| 2.5 \| \| \| *pncA* c.390_391insG \|  \|  \|  \|  \|  \|  \|  \|  \|  \| 1 \| 2.5 \| \| \| *pncA* c.392_393insC \|  \|  \|  \|  \|  \|  \|  \|  \|  \| 1 \| 2.5 \| \| \| *pncA* c.394delG \|  \|  \|  \|  \|  \|  \|  \|  \|  \| 1 \| 2.5 \| \| \| *pncA* c.417_425insCTTGTGTG \|  \|  \|  \|  \|  \|  \|  \|  \|  \| 1 \| 2.5 \| \| \| *pncA* c.437_439insGC \|  \|  \|  \|  \|  \|  \|  \|  \|  \| 1 \| 2.5 \| \| \| *pncA* deletion \|  \|  \|  \|  \|  \|  \|  \|  \|  \| 2 \| 5.0 \| \| \| WT \|  \|  \|  \|  \|  \|  \|  \|  \|  \| 1 \| 2.5 \| \| \| Susceptible  (160, 80.0%) \| *pncA* L116P \|  \|  \|  \|  \|  \|  \|  \|  \|  \| 1 \| 0.6 \| \| \| *pncA* T168P \|  \|  \|  \|  \|  \|  \|  \|  \|  \| 1 \| 0.6 \| \| \| WT \|  \|  \|  \|  \|  \|  \|  \|  \|  \| 158 \| 98.8 \| \| \| STR \|  \|  \| ≤0.25 \| 0.5 \| 1 \| 2 \| 4 \| 8 \| 16 \| 32 \| >32 \|  \|  \| \| \|  \| Resistant  (60, 30.0%) \| *rpsL* K43R \|  \|  \|  \|  \|  \|  \|  \|  \| 31 \| 31 \| 51.7 \| \| \| *rpsL* K88R \|  \| 1 \| 2 \|  \|  \| 2 \| 1 \| 1 \| 2 \| 9 \| 15.0 \| \| \| *rpsL* K88M \|  \| 1 \|  \|  \|  \|  \|  \|  \|  \| 1 \| 1.7 \| \| \| *rrs* a514c \| 2 \| 2 \|  \|  \| 1 \|  \| 1 \|  \| 1 \| 7 \| 11.7 \| \| \| *rrs* a906g \| 1 \|  \|  \| 1 \|  \|  \|  \| 1 \|  \| 3 \| 5.0 \| \| \| *rrs* c517t \|  \|  \|  \|  \| 1 \|  \|  \| 1 \|  \| 2 \| 3.3 \| \| \| *gid* G71R \| 1 \|  \|  \|  \|  \|  \|  \|  \|  \| 1 \| 1.7 \| \| \| *gid* R96C \| 1 \|  \|  \|  \|  \|  \|  \|  \|  \| 1 \| 1.7 \| \| \| *gid* A138V \|  \|  \|  \| 1 \|  \|  \|  \|  \|  \| 1 \| 1.7 \| \| \| *gid* c.114delC \|  \|  \| 1 \|  \|  \|  \|  \|  \|  \| 1 \| 1.7 \| \| \| *gid* c.524delG \|  \| 1 \|  \|  \|  \|  \|  \|  \|  \| 1 \| 1.7 \| \| \| WT \|  \|  \|  \|  \|  \|  \| 1 \|  \| 1 \| 2 \| 3.3 \| \| \| Susceptible  (140, 70.0%) \| *rpsL* K88Q \|  \|  \| 1 \|  \|  \|  \|  \|  \|  \| 1 \| 0.7 \| \| \| *rrs* a514c \|  \|  \| 3 \|  \|  \|  \|  \|  \|  \| 3 \| 2.1 \| \| \| *rrs* a906g \| 1 \| 1 \|  \|  \|  \|  \|  \|  \|  \| 2 \| 1.4 \| \| \| *rrs* c517t \|  \| 1 \| 1 \| 1 \|  \|  \|  \|  \|  \| 3 \| 2.1 \| \| \| *gid* A138V \|  \|  \|  \| 1 \|  \|  \|  \|  \|  \| 1 \| 0.7 \| \| \| *gid* c.350_351insG \|  \| 1 \| 1 \|  \|  \| 1 \|  \|  \|  \| 3 \| 2.1 \| \| \| *gid* c.350delG \|  \| 1 \| 1 \| 1 \|  \| 1 \|  \|  \|  \| 4 \| 2.9 \| \| \| *gid* c.101delG \|  \| 1 \|  \| 1 \| 1 \|  \|  \|  \|  \| 3 \| 2.1 \| \| \| *gid* c.114delC \| 1 \|  \|  \|  \|  \|  \|  \|  \|  \| 1 \| 0.7 \| \| \| *gid* c.516_534insGCTCACGACGAAGTACGG \|  \| 1 \|  \|  \|  \|  \|  \|  \|  \| 1 \| 0.7 \| \| \| WT \| 56 \| 35 \| 18 \| 6 \| 2 \|  \| 1 \|  \|  \| 118 \| 84.2 \| \| \| OFX \|  \|  \| ≤0.25 \| 0.5 \| 1 \| 2 \| 4 \| 8 \| 16 \| 32 \| >32 \|  \|  \| \| \|  \| Resistant  (22, 11.0%) \| *gyrA* D94G \|  \|  \|  \|  \|  \| 3 \| 6 \| 4 \|  \| 13 \| 59.1 \| \| \| *gyrA* D94G, *gyrA* A90V \|  \|  \|  \|  \|  \|  \|  \|  \| 1 \| 1 \| 4.5 \| \| \| *gyrA* D94A \|  \|  \|  \|  \|  \|  \|  \| 1 \|  \| 1 \| 4.5 \| \| \| *gyrA* D94H \|  \|  \|  \|  \|  \|  \|  \| 1 \|  \| 1 \| 4.5 \| \| \| *gyrA* D94N \|  \|  \|  \|  \|  \|  \| 1 \| 1 \|  \| 2 \| 9.1 \| \| \| *gyrA* G88C \|  \|  \|  \|  \|  \|  \| 1 \|  \|  \| 1 \| 4.5 \| \| \| *gyrA* A90V \|  \|  \|  \|  \|  \| 1 \|  \|  \|  \| 1 \| 4.5 \| \| \| *gyrA* S91P \|  \|  \|  \|  \|  \| 1 \|  \|  \|  \| 1 \| 4.5 \| \| \| *gyrB* G522S \|  \|  \|  \|  \|  \| 1 \|  \|  \|  \| 1 \| 4.5 \| \| \| Susceptible  (178, 89.0%) \| *gyrA* D94G \|  \|  \| 1 \|  \|  \|  \|  \|  \|  \| 1 \| 0.6 \| \| \| WT \| 13 \| 47 \| 83 \| 21 \| 8 \| 1 \| 2 \| 1 \| 1 \| 177 \| 99.4 \| \| \| MFX \|  \|  \| ≤0.06 \| 0.12 \| 0.25 \| 0.5 \| 1 \| 2 \| 4 \| 8 \| >8 \|  \|  \| \| \|  \| Resistant  (22, 11.0%) \| *gyrA* D94G \|  \|  \|  \|  \|  \|  \| 4 \| 8 \| 1 \| 13 \| 59.1 \| \| \| *gyrA* D94G, gyrA A90V \|  \|  \|  \|  \|  \|  \|  \| 1 \|  \| 1 \| 4.5 \| \| \| *gyrA* D94A \|  \|  \|  \|  \|  \|  \|  \| 1 \|  \| 1 \| 4.5 \| \| \| *gyrA* D94H \|  \|  \|  \|  \|  \|  \|  \| 1 \|  \| 1 \| 4.5 \| \| \| *gyrA* D94N \|  \|  \|  \|  \|  \|  \|  \| 1 \| 1 \| 2 \| 9.1 \| \| \| *gyrA* G88C \|  \|  \|  \|  \|  \|  \|  \| 1 \|  \| 1 \| 4.5 \| \| \| *gyrA* A90V \|  \|  \|  \|  \|  \|  \| 1 \|  \|  \| 1 \| 4.5 \| \| \| *gyrA* S91P \|  \|  \|  \|  \|  \| 1 \|  \|  \|  \| 1 \| 4.5 \| \| \| *gyrB* G522S \|  \|  \|  \| 1 \|  \|  \|  \|  \|  \| 1 \| 4.5 \| \| \| Susceptible  (178, 89.0%) \| gyrA D94G \|  \|  \| 1 \|  \|  \|  \|  \|  \|  \| 1 \| 0.6 \| \| \| WT \| 24 \| 46 \| 47 \| 38 \| 15 \| 3 \|  \| 3 \| 1 \| 177 \| 99.4 \| \| \| KM \|  \|  \| ≤0.6 \| 1.2 \| 2.5 \| 5 \| 10 \| 20 \| 40 \| >40 \|  \|  \|  \| \| \|  \| Resistant  (11, 5.5%) \| *rrs* a1401g \|  \|  \|  \|  \|  \|  \|  \| 7 \|  \| 7 \| 63.6 \| \| \| *eis* g-10a \|  \|  \|  \|  \| 1 \|  \|  \|  \|  \| 1 \| 9.1 \| \| \| *eis* g-37t \|  \|  \|  \| 1 \|  \|  \|  \|  \|  \| 1 \| 9.1 \| \| \| WT \|  \|  \|  \| 1 \|  \|  \|  \| 1 \|  \| 2 \| 18.2 \| \| \| Susceptible  (189, 94.5%) \| *eis* c-12t \|  \| 1 \| 2 \| 3 \|  \|  \|  \|  \|  \| 6 \| 3.2 \| \| \| WT \| 49 \| 90 \| 37 \| 4 \| 2 \|  \|  \| 1 \|  \| 183 \| 96.8 \| \| \| AMK \|  \|  \| ≤0.12 \| 0.25 \| 0.5 \| 1 \| 2 \| 4 \| 8 \| 16 \| >16 \|  \|  \| \| \|  \| Resistant  (8, 4.0%) \| *rrs* a1401g \|  \|  \|  \|  \|  \|  \|  \|  \| 7 \| 7 \| 87.5 \| \| \| WT \|  \|  \|  \|  \|  \|  \|  \|  \| 1 \| 1 \| 12.5 \| \| \| Susceptible  (192, 96.0%) \| WT \| 33 \| 64 \| 77 \| 15 \| 1 \|  \| 1 \| 1 \|  \| 192 \| 100 \| \| \| CM \|  \|  \|  \|  \|  \|  \|  \|  \|  \|  \|  \|  \|  \| \| \|  \| Resistant  (6, 3.0%) \| *rrs* a1401g \|  \|  \|  \|  \|  \|  \|  \|  \|  \| 4 \| 66.7 \| \| \| *rrs* c.623_624insG \|  \|  \|  \|  \|  \|  \|  \|  \|  \| 1 \| 16.7 \| \| \| WT \|  \|  \|  \|  \|  \|  \|  \|  \|  \| 1 \| 16.7 \| \| \| Susceptible  (194, 97.0%) \| *rrs* a1401g \|  \|  \|  \|  \|  \|  \|  \|  \|  \| 3 \| 1.5 \| \| \| WT \|  \|  \|  \|  \|  \|  \|  \|  \|  \| 191 \| 98.5 \| \| \| ETO \|  \|  \| ≤0.3 \| 0.6 \| 1.2 \| 2.5 \| 5 \| 10 \| 20 \| 40 \| >40 \|  \|  \| \| \|  \| Resistant  (34, 17.0%) \| *fabG1* c-15t \|  \| 3 \| 4 \| 5 \| 3 \| 1 \| 1 \|  \| 2 \| 19 \| 55.9 \| \| \| *fabG1* c-15t, *inhA* I194T \|  \| 1 \|  \|  \|  \|  \|  \|  \| 3 \| 4 \| 11.8 \| \| \| *fabG1* c-15t, *ethR* A95T \|  \|  \| 1 \|  \|  \|  \|  \|  \|  \| 1 \| 2.9 \| \| \| *fabG1* c-15t, *ethA* Q165P \|  \|  \|  \|  \|  \|  \|  \|  \| 1 \| 1 \| 2.9 \| \| \| *fabG1* t-8c, *ethA* c.1321delC \|  \|  \|  \|  \|  \| 1 \|  \|  \|  \| 1 \| 2.9 \| \| \| *fabG1* g-17t, *ethA* c.70delC \|  \|  \|  \|  \|  \|  \|  \| 1 \|  \| 1 \| 2.9 \| \| \| *fabG1* L203L \|  \|  \| 1 \|  \| 1 \|  \|  \|  \|  \| 2 \| 5.9 \| \| \| *ethA* c.320_321insC \|  \| 1 \|  \|  \|  \|  \|  \|  \|  \| 1 \| 2.9 \| \| \| *ethA* c.1276delG \|  \|  \|  \|  \| 1 \|  \|  \|  \|  \| 1 \| 2.9 \| \| \| *ethA* insertion/deletion \|  \|  \|  \|  \| 1 \| 1 \|  \|  \|  \| 2 \| 5.9 \| \| \| WT \|  \|  \| 1 \|  \|  \|  \|  \|  \|  \| 1 \| 2.9 \| \| \| Susceptible  (166, 83.0%) \| *fabG1* c-15t \| 2 \|  \| 1 \| 5 \| 5 \|  \|  \|  \| 1 \| 14 \| 8.4 \| \| \| *fabG1* c-15t, *inhA* I194T \|  \|  \|  \| 2 \|  \|  \|  \|  \|  \| 2 \| 1.2 \| \| \| *fabG1* c-15t, *ethR* A95T \|  \| 1 \| 2 \| 1 \|  \|  \|  \|  \|  \| 4 \| 2.4 \| \| \| *fabG1* t-8c \|  \| 2 \| 1 \|  \|  \|  \|  \|  \|  \| 3 \| 1.8 \| \| \| *inhA* S94A \|  \|  \| 1 \|  \|  \|  \|  \|  \|  \| 1 \| 0.6 \| \| \| *ethA* c.298delT \|  \| 1 \|  \|  \|  \|  \|  \|  \|  \| 1 \| 0.6 \| \| \| *ethA* c.551_555insCTGG \|  \|  \| 1 \|  \|  \|  \|  \|  \|  \| 1 \| 0.6 \| \| \| *ethA* c.923_924insG \|  \|  \|  \|  \|  \| 1 \|  \|  \|  \| 1 \| 0.6 \| \| \| *ethA* insertion/deletion \|  \| 2 \| 1 \|  \|  \|  \|  \|  \| 1 \| 4 \| 2.4 \| \| \| WT \| 56 \| 49 \| 24 \| 6 \|  \|  \|  \|  \|  \| 135 \| 81.3 \| \| \| PAS \|  \|  \| ≤0.5 \| 1 \| 2 \| 4 \| 8 \| 16 \| 32 \| 64 \| >64 \|  \|  \| \| \|  \| Resistant  (6, 3.0%) \| *folC* E40G \|  \|  \|  \|  \|  \|  \|  \| 1 \|  \| 1 \| 16.7 \| \| \| *thyA* L38S \|  \|  \| 1 \|  \|  \|  \|  \|  \|  \| 1 \| 16.7 \| \| \| *thyA* L218P \|  \|  \|  \| 1 \|  \|  \|  \|  \| 1 \| 2 \| 33.3 \| \| \| *thyA* R235W \|  \|  \|  \|  \|  \|  \|  \|  \| 1 \| 1 \| 16.7 \| \| \| *thyA* Y251stop \|  \|  \|  \|  \|  \|  \|  \|  \| 1 \| 1 \| 16.7 \| \| \| Susceptible  (194, 97.0%) \| *folC* S150G \|  \|  \|  \| 1 \|  \|  \|  \|  \|  \| 1 \| 0.5 \| \| \| WT \| 175 \| 10 \| 2 \| 1 \| 2 \|  \|  \| 1 \| 2 \| 193 \| 99.5 \| \| \| Abbreviations: RIF, rifampicin; INH, isoniazid; EMB, ethambutol; PZA, pyrazinamide; STR, streptomycin; OFX, ofloxacin; MFX, moxifloxacin; KM, kanamycin; AMK, amikacin; CM, capreomycin; ETO, ethionamide; PAS, para-aminosalicylic acid; pDST, phenotypic drug susceptibility testing; WT, wild type; ins, insertion; del, deletion. \| \| \| \| \| \| \| \| \| \| \| \| \| \| |  |
| --- | --- | --- | --- | --- | --- | --- | --- | --- | --- | --- | --- | --- | --- | --- | --- | --- | --- | --- | --- | --- | --- | --- | --- | --- | --- | --- | --- | --- | --- | --- | --- | --- | --- | --- | --- | --- | --- | --- | --- | --- | --- | --- | --- | --- | --- | --- | --- | --- | --- | --- | --- | --- | --- | --- | --- | --- | --- | --- | --- | --- | --- | --- | --- | --- | --- | --- | --- | --- | --- | --- | --- | --- | --- | --- | --- | --- | --- | --- | --- | --- | --- | --- | --- | --- | --- | --- | --- | --- | --- | --- | --- | --- | --- | --- | --- | --- | --- | --- | --- | --- | --- | --- | --- | --- | --- | --- | --- | --- | --- | --- | --- | --- | --- | --- | --- | --- | --- | --- | --- | --- | --- | --- | --- | --- | --- | --- | --- | --- | --- | --- | --- | --- | --- | --- | --- | --- | --- | --- | --- | --- | --- | --- | --- | --- | --- | --- | --- | --- | --- | --- | --- | --- | --- | --- | --- | --- | --- | --- | --- | --- | --- | --- | --- | --- | --- | --- | --- | --- | --- | --- | --- | --- | --- | --- | --- | --- | --- | --- | --- | --- | --- | --- | --- | --- | --- | --- | --- | --- | --- | --- | --- | --- | --- | --- | --- | --- | --- | --- | --- | --- | --- | --- | --- | --- | --- | --- | --- | --- | --- | --- | --- | --- | --- | --- | --- | --- | --- | --- | --- | --- | --- | --- | --- | --- | --- | --- | --- | --- | --- | --- | --- | --- | --- | --- | --- | --- | --- | --- | --- | --- | --- | --- | --- | --- | --- | --- | --- | --- | --- | --- | --- | --- | --- | --- | --- | --- | --- | --- | --- | --- | --- | --- | --- | --- | --- | --- | --- | --- | --- | --- | --- | --- | --- | --- | --- | --- | --- | --- | --- | --- | --- | --- | --- | --- | --- | --- | --- | --- | --- | --- | --- | --- | --- | --- | --- | --- | --- | --- | --- | --- | --- | --- | --- | --- | --- | --- | --- | --- | --- | --- | --- | --- | --- | --- | --- | --- | --- | --- | --- | --- | --- | --- | --- | --- | --- | --- | --- | --- | --- | --- | --- | --- | --- | --- | --- | --- | --- | --- | --- | --- | --- | --- | --- | --- | --- | --- | --- | --- | --- | --- | --- | --- | --- | --- | --- | --- | --- | --- | --- | --- | --- | --- | --- | --- | --- | --- | --- | --- | --- | --- | --- | --- | --- | --- | --- | --- | --- | --- | --- | --- | --- | --- | --- | --- | --- | --- | --- | --- | --- | --- | --- | --- | --- | --- | --- | --- | --- | --- | --- | --- | --- | --- | --- | --- | --- | --- | --- | --- | --- | --- | --- | --- | --- | --- | --- | --- | --- | --- | --- | --- | --- | --- | --- | --- | --- | --- | --- | --- | --- | --- | --- | --- | --- | --- | --- | --- | --- | --- | --- | --- | --- | --- | --- | --- | --- | --- | --- | --- | --- | --- | --- | --- | --- | --- | --- | --- | --- | --- | --- | --- | --- | --- | --- | --- | --- | --- | --- | --- | --- | --- | --- | --- | --- | --- | --- | --- | --- | --- | --- | --- | --- | --- | --- | --- | --- | --- | --- | --- | --- | --- | --- | --- | --- | --- | --- | --- | --- | --- | --- | --- | --- | --- | --- | --- | --- | --- | --- | --- | --- | --- | --- | --- | --- | --- | --- | --- | --- | --- | --- | --- | --- | --- | --- | --- | --- | --- | --- | --- | --- | --- | --- | --- | --- | --- | --- | --- | --- | --- | --- | --- | --- | --- | --- | --- | --- | --- | --- | --- | --- | --- | --- | --- | --- | --- | --- | --- | --- | --- | --- | --- | --- | --- | --- | --- | --- | --- | --- | --- | --- | --- | --- | --- | --- | --- | --- | --- | --- | --- | --- | --- | --- | --- | --- | --- | --- | --- | --- | --- | --- | --- | --- | --- | --- | --- | --- | --- | --- | --- | --- | --- | --- | --- | --- | --- | --- | --- | --- | --- | --- | --- | --- | --- | --- | --- | --- | --- | --- | --- | --- | --- | --- | --- | --- | --- | --- | --- | --- | --- | --- | --- | --- | --- | --- | --- | --- | --- | --- | --- | --- | --- | --- | --- | --- | --- | --- | --- | --- | --- | --- | --- | --- | --- | --- | --- | --- | --- | --- | --- | --- | --- | --- | --- | --- | --- | --- | --- | --- | --- | --- | --- | --- | --- | --- | --- | --- | --- | --- | --- | --- | --- | --- | --- | --- | --- | --- | --- | --- | --- | --- | --- | --- | --- | --- | --- | --- | --- | --- | --- | --- | --- | --- | --- | --- | --- | --- | --- | --- | --- | --- | --- | --- | --- | --- | --- | --- | --- | --- | --- | --- | --- | --- | --- | --- | --- | --- | --- | --- | --- | --- | --- | --- | --- | --- | --- | --- | --- | --- | --- | --- | --- | --- | --- | --- | --- | --- | --- | --- | --- | --- | --- | --- | --- | --- | --- | --- | --- | --- | --- | --- | --- | --- | --- | --- | --- | --- | --- | --- | --- | --- | --- | --- | --- | --- | --- | --- | --- | --- | --- | --- | --- | --- | --- | --- | --- | --- | --- | --- | --- | --- | --- | --- | --- | --- | --- | --- | --- | --- | --- | --- | --- | --- | --- | --- | --- | --- | --- | --- | --- | --- | --- | --- | --- | --- | --- | --- | --- | --- | --- | --- | --- | --- | --- | --- | --- | --- | --- | --- | --- | --- | --- | --- | --- | --- | --- | --- | --- | --- | --- | --- | --- | --- | --- | --- | --- | --- | --- | --- | --- | --- | --- | --- | --- | --- | --- | --- | --- | --- | --- | --- | --- | --- | --- | --- | --- | --- | --- | --- | --- | --- | --- | --- | --- | --- | --- | --- | --- | --- | --- | --- | --- | --- | --- | --- | --- | --- | --- | --- | --- | --- | --- | --- | --- | --- | --- | --- | --- | --- | --- | --- | --- | --- | --- | --- | --- | --- | --- | --- | --- | --- | --- | --- | --- | --- | --- | --- | --- | --- | --- | --- | --- | --- | --- | --- | --- | --- | --- | --- | --- | --- | --- | --- | --- | --- | --- | --- | --- | --- | --- | --- | --- | --- | --- | --- | --- | --- | --- | --- | --- | --- | --- | --- | --- | --- | --- | --- | --- | --- | --- | --- | --- | --- | --- | --- | --- | --- | --- | --- | --- | --- | --- | --- | --- | --- | --- | --- | --- | --- | --- | --- | --- | --- | --- | --- | --- | --- | --- | --- | --- | --- | --- | --- | --- | --- | --- | --- | --- | --- | --- | --- | --- | --- | --- | --- | --- | --- | --- | --- | --- | --- | --- | --- | --- | --- | --- | --- | --- | --- | --- | --- | --- | --- | --- | --- | --- | --- | --- | --- | --- | --- | --- | --- | --- | --- | --- | --- | --- | --- | --- | --- | --- | --- | --- | --- | --- | --- | --- | --- | --- | --- | --- | --- | --- | --- | --- | --- | --- | --- | --- | --- | --- | --- | --- | --- | --- | --- | --- | --- | --- | --- | --- | --- | --- | --- | --- | --- | --- | --- | --- | --- | --- | --- | --- | --- | --- | --- | --- | --- | --- | --- | --- | --- | --- | --- | --- | --- | --- | --- | --- | --- | --- | --- | --- | --- | --- | --- | --- | --- | --- | --- | --- | --- | --- | --- | --- | --- | --- | --- | --- | --- | --- | --- | --- | --- | --- | --- | --- | --- | --- | --- | --- | --- | --- | --- | --- | --- | --- | --- | --- | --- | --- | --- | --- | --- | --- | --- | --- | --- | --- | --- | --- | --- | --- | --- | --- | --- | --- | --- | --- | --- | --- | --- | --- | --- | --- | --- | --- | --- | --- | --- | --- | --- | --- | --- | --- | --- | --- | --- | --- | --- | --- | --- | --- | --- | --- | --- | --- | --- | --- | --- | --- | --- | --- | --- | --- | --- | --- | --- | --- | --- | --- | --- | --- | --- | --- | --- | --- | --- | --- | --- | --- | --- | --- | --- | --- | --- | --- | --- | --- | --- | --- | --- | --- | --- | --- | --- | --- | --- | --- | --- | --- | --- | --- | --- | --- | --- | --- | --- | --- | --- | --- | --- | --- | --- | --- | --- | --- | --- | --- | --- | --- | --- | --- | --- | --- | --- | --- | --- | --- | --- | --- | --- | --- | --- | --- | --- | --- | --- | --- | --- | --- | --- | --- | --- | --- | --- | --- | --- | --- | --- | --- | --- | --- | --- | --- | --- | --- | --- | --- | --- | --- | --- | --- | --- | --- | --- | --- | --- | --- | --- | --- | --- | --- | --- | --- | --- | --- | --- | --- | --- | --- | --- | --- | --- | --- | --- | --- | --- | --- | --- | --- | --- | --- | --- | --- | --- | --- | --- | --- | --- | --- | --- | --- | --- | --- | --- | --- | --- | --- | --- | --- | --- | --- | --- | --- | --- | --- | --- | --- | --- | --- | --- | --- | --- | --- | --- | --- | --- | --- | --- | --- | --- | --- | --- | --- | --- | --- | --- | --- | --- | --- | --- | --- | --- | --- | --- | --- | --- | --- | --- | --- | --- | --- | --- | --- | --- | --- | --- | --- | --- | --- | --- | --- | --- | --- | --- | --- | --- | --- | --- | --- | --- | --- | --- | --- | --- | --- | --- | --- | --- | --- | --- | --- | --- | --- | --- | --- | --- | --- | --- | --- | --- | --- | --- | --- | --- | --- | --- | --- | --- | --- | --- | --- | --- | --- | --- | --- | --- | --- | --- | --- | --- | --- | --- | --- | --- | --- | --- | --- | --- | --- | --- | --- | --- | --- | --- | --- | --- | --- | --- | --- | --- | --- | --- | --- | --- | --- | --- | --- | --- | --- | --- | --- | --- | --- | --- | --- | --- | --- | --- | --- | --- | --- | --- | --- | --- | --- | --- | --- | --- | --- | --- | --- | --- | --- | --- | --- | --- | --- | --- | --- | --- | --- | --- | --- | --- | --- | --- | --- | --- | --- | --- | --- | --- | --- | --- | --- | --- | --- | --- | --- | --- | --- | --- | --- | --- | --- | --- | --- | --- | --- | --- | --- | --- | --- | --- | --- | --- | --- | --- | --- | --- | --- | --- | --- | --- | --- | --- | --- | --- | --- | --- | --- | --- | --- | --- | --- | --- | --- | --- | --- | --- | --- | --- | --- | --- | --- | --- | --- | --- | --- | --- | --- | --- | --- | --- | --- | --- | --- | --- | --- | --- | --- | --- | --- | --- | --- | --- | --- | --- | --- | --- | --- | --- | --- | --- | --- | --- | --- | --- | --- | --- | --- | --- | --- | --- | --- | --- | --- | --- | --- | --- | --- | --- | --- | --- | --- | --- | --- | --- | --- | --- | --- | --- | --- | --- | --- | --- | --- | --- | --- | --- | --- | --- | --- | --- | --- | --- | --- | --- | --- | --- | --- | --- | --- | --- | --- | --- | --- | --- | --- | --- | --- | --- | --- | --- | --- | --- | --- | --- | --- | --- | --- | --- | --- | --- | --- | --- | --- | --- | --- | --- | --- | --- | --- | --- | --- | --- | --- | --- | --- | --- | --- | --- | --- | --- | --- | --- | --- | --- | --- | --- | --- | --- | --- | --- | --- | --- | --- | --- | --- | --- | --- | --- | --- | --- | --- | --- | --- | --- | --- | --- | --- | --- | --- | --- | --- | --- | --- | --- | --- | --- | --- | --- | --- | --- | --- | --- | --- | --- | --- | --- | --- | --- | --- | --- | --- | --- | --- | --- | --- | --- | --- | --- | --- | --- | --- | --- | --- | --- | --- | --- | --- | --- | --- | --- | --- | --- | --- | --- | --- | --- | --- | --- | --- | --- | --- | --- | --- | --- | --- | --- | --- | --- | --- | --- | --- | --- | --- | --- | --- | --- | --- | --- | --- | --- | --- | --- | --- | --- | --- | --- | --- | --- | --- | --- | --- | --- | --- | --- | --- | --- | --- | --- | --- | --- | --- | --- | --- | --- | --- | --- | --- | --- | --- | --- | --- | --- | --- | --- | --- | --- | --- | --- | --- | --- | --- | --- | --- | --- | --- | --- | --- | --- | --- | --- | --- | --- | --- | --- | --- | --- | --- | --- | --- | --- | --- | --- | --- | --- | --- | --- | --- | --- | --- | --- | --- | --- | --- | --- | --- | --- | --- | --- | --- | --- | --- | --- | --- | --- | --- | --- | --- | --- | --- | --- | --- | --- | --- | --- | --- | --- | --- | --- | --- | --- | --- | --- | --- | --- | --- | --- | --- | --- | --- | --- | --- | --- | --- | --- | --- | --- | --- | --- | --- | --- | --- | --- | --- | --- | --- | --- | --- | --- | --- | --- | --- | --- | --- | --- | --- | --- | --- | --- | --- | --- | --- | --- | --- | --- | --- | --- | --- | --- | --- | --- | --- | --- | --- | --- | --- | --- | --- | --- | --- | --- | --- | --- | --- | --- | --- | --- | --- | --- | --- | --- | --- | --- | --- | --- | --- | --- | --- | --- | --- | --- | --- | --- | --- | --- | --- | --- | --- | --- | --- | --- | --- | --- | --- | --- | --- | --- | --- | --- | --- | --- | --- | --- | --- | --- | --- | --- | --- | --- | --- | --- | --- | --- | --- | --- | --- | --- | --- | --- | --- | --- | --- | --- | --- | --- | --- | --- | --- | --- | --- | --- | --- | --- | --- | --- | --- | --- | --- | --- | --- | --- | --- | --- | --- | --- | --- | --- | --- | --- | --- | --- | --- | --- | --- | --- | --- | --- | --- | --- | --- | --- | --- | --- | --- | --- | --- | --- | --- | --- | --- | --- | --- | --- | --- | --- | --- | --- | --- | --- | --- | --- | --- | --- | --- | --- | --- | --- | --- | --- | --- | --- | --- | --- | --- | --- | --- | --- | --- | --- | --- | --- | --- | --- | --- | --- | --- | --- | --- | --- | --- | --- | --- | --- | --- | --- | --- | --- | --- | --- | --- | --- | --- | --- | --- | --- | --- | --- | --- | --- | --- | --- | --- | --- | --- | --- | --- | --- | --- | --- | --- | --- | --- | --- | --- | --- | --- | --- | --- | --- | --- | --- | --- | --- | --- | --- | --- | --- | --- | --- | --- | --- | --- | --- | --- | --- | --- | --- | --- | --- | --- | --- | --- | --- | --- | --- | --- | --- | --- | --- | --- | --- | --- | --- | --- | --- | --- | --- | --- | --- | --- | --- | --- | --- | --- | --- | --- | --- | --- | --- | --- | --- | --- | --- | --- | --- | --- | --- | --- | --- | --- | --- | --- | --- | --- | --- | --- | --- | --- | --- | --- | --- | --- | --- | --- | --- | --- | --- | --- | --- | --- | --- | --- | --- | --- | --- | --- | --- | --- | --- | --- | --- | --- | --- | --- | --- | --- | --- | --- | --- | --- | --- | --- | --- | --- | --- | --- | --- | --- | --- | --- | --- | --- | --- | --- | --- | --- | --- | --- | --- | --- | --- | --- | --- | --- | --- | --- | --- | --- | --- | --- | --- | --- | --- | --- | --- | --- | --- | --- | --- | --- | --- | --- | --- | --- | --- | --- | --- | --- | --- | --- | --- | --- | --- | --- | --- | --- | --- | --- | --- | --- | --- | --- | --- | --- | --- | --- | --- | --- | --- | --- | --- | --- | --- | --- | --- | --- | --- | --- | --- | --- | --- | --- | --- | --- | --- | --- | --- | --- | --- | --- | --- | --- | --- | --- | --- | --- | --- | --- | --- | --- | --- | --- | --- | --- | --- | --- | --- | --- | --- | --- | --- | --- | --- | --- | --- | --- | --- | --- | --- | --- | --- | --- | --- | --- | --- | --- | --- | --- | --- | --- | --- | --- | --- | --- | --- | --- | --- | --- | --- | --- | --- | --- | --- | --- | --- | --- | --- | --- | --- | --- | --- | --- | --- | --- | --- | --- | --- | --- | --- | --- | --- | --- | --- | --- | --- | --- | --- | --- | --- | --- | --- | --- | --- | --- | --- | --- | --- | --- | --- | --- | --- | --- | --- | --- | --- | --- | --- | --- | --- | --- | --- | --- | --- | --- | --- | --- | --- | --- | --- | --- | --- | --- | --- | --- | --- | --- | --- | --- | --- | --- | --- | --- | --- | --- | --- | --- | --- | --- | --- | --- | --- | --- | --- | --- | --- | --- | --- | --- | --- | --- | --- | --- | --- | --- | --- | --- | --- | --- | --- | --- | --- | --- | --- | --- | --- | --- | --- | --- | --- | --- | --- | --- | --- | --- | --- | --- | --- | --- | --- | --- | --- | --- | --- | --- | --- | --- | --- | --- | --- | --- | --- | --- | --- | --- | --- | --- | --- | --- | --- | --- | --- | --- | --- | --- | --- | --- | --- | --- | --- | --- | --- | --- | --- | --- | --- | --- | --- | --- | --- | --- | --- | --- | --- | --- | --- | --- | --- | --- | --- | --- | --- | --- | --- | --- | --- | --- | --- | --- | --- | --- | --- | --- | --- | --- | --- | --- | --- | --- | --- | --- | --- | --- | --- | --- | --- | --- | --- | --- | --- | --- | --- | --- | --- | --- | --- | --- | --- | --- | --- | --- | --- | --- | --- | --- | --- | --- | --- | --- | --- | --- | --- | --- | --- | --- | --- | --- | --- | --- | --- | --- | --- | --- | --- | --- | --- | --- | --- | --- | --- | --- | --- | --- | --- | --- | --- | --- | --- | --- | --- | --- | --- | --- | --- | --- | --- | --- | --- | --- | --- | --- | --- | --- | --- | --- | --- | --- | --- | --- | --- | --- | --- | --- | --- | --- | --- | --- | --- | --- | --- | --- | --- | --- | --- | --- | --- | --- | --- | --- | --- | --- | --- | --- | --- | --- | --- | --- | --- | --- | --- | --- | --- | --- | --- | --- | --- | --- | --- | --- | --- | --- | --- | --- | --- | --- | --- | --- | --- | --- | --- | --- | --- | --- | --- | --- | --- | --- | --- | --- | --- | --- | --- | --- | --- | --- | --- | --- | --- | --- | --- | --- | --- | --- | --- | --- | --- | --- | --- | --- | --- | --- | --- | --- | --- | --- | --- | --- | --- | --- | --- | --- | --- | --- | --- | --- | --- | --- | --- | --- | --- | --- | --- | --- | --- | --- | --- | --- | --- | --- | --- | --- | --- | --- | --- | --- | --- | --- | --- | --- | --- | --- | --- | --- | --- | --- | --- | --- | --- | --- | --- | --- | --- | --- | --- | --- | --- | --- | --- | --- | --- | --- | --- | --- | --- | --- | --- | --- | --- | --- | --- | --- | --- | --- | --- | --- | --- | --- | --- | --- | --- | --- | --- | --- | --- | --- | --- | --- | --- | --- | --- | --- | --- | --- | --- | --- | --- | --- | --- | --- | --- | --- | --- | --- | --- | --- | --- | --- | --- | --- | --- | --- | --- | --- | --- | --- | --- | --- | --- | --- | --- | --- | --- | --- | --- | --- | --- | --- | --- | --- | --- | --- | --- | --- | --- | --- | --- | --- | --- | --- | --- | --- | --- | --- | --- | --- | --- | --- | --- | --- | --- | --- | --- | --- | --- | --- | --- | --- | --- | --- | --- | --- | --- | --- | --- | --- | --- | --- | --- | --- | --- | --- | --- | --- | --- | --- | --- | --- | --- | --- | --- | --- | --- | --- | --- | --- | --- | --- | --- | --- | --- | --- | --- | --- | --- | --- | --- | --- | --- | --- | --- | --- | --- | --- | --- | --- | --- | --- | --- | --- | --- | --- | --- | --- | --- | --- | --- | --- | --- | --- | --- | --- | --- | --- | --- | --- | --- | --- | --- | --- | --- | --- | --- | --- | --- | --- | --- | --- | --- | --- | --- | --- | --- | --- | --- | --- | --- | --- | --- | --- | --- | --- | --- | --- | --- | --- | --- | --- | --- | --- | --- | --- | --- | --- | --- | --- | --- | --- | --- | --- | --- | --- | --- | --- | --- | --- | --- | --- | --- | --- | --- | --- | --- | --- | --- | --- | --- | --- | --- | --- | --- | --- | --- | --- | --- | --- | --- | --- | --- | --- | --- | --- | --- | --- | --- | --- | --- | --- | --- | --- | --- | --- | --- | --- | --- | --- | --- | --- | --- | --- | --- | --- | --- | --- | --- | --- | --- | --- | --- | --- | --- | --- | --- | --- | --- | --- | --- | --- | --- | --- | --- | --- | --- | --- | --- | --- | --- | --- | --- | --- | --- | --- | --- | --- | --- | --- | --- | --- | --- | --- | --- | --- | --- | --- | --- | --- | --- | --- | --- | --- | --- | --- | --- | --- | --- | --- | --- | --- | --- | --- | --- | --- | --- | --- | --- | --- | --- | --- | --- | --- | --- | --- | --- | --- | --- | --- | --- | --- | --- | --- | --- | --- | --- | --- | --- | --- | --- | --- | --- | --- | --- | --- | --- | --- | --- | --- | --- | --- | --- | --- | --- | --- | --- | --- | --- | --- | --- | --- | --- | --- | --- | --- | --- | --- | --- | --- | --- | --- | --- | --- | --- | --- | --- | --- | --- | --- | --- | --- | --- | --- | --- | --- | --- | --- | --- | --- | --- | --- | --- | --- | --- | --- | --- | --- | --- | --- | --- | --- | --- | --- | --- | --- | --- | --- | --- | --- | --- | --- | --- | --- | --- | --- | --- | --- | --- | --- | --- | --- | --- | --- | --- | --- | --- | --- | --- | --- | --- | --- | --- | --- | --- | --- | --- | --- | --- | --- | --- | --- | --- | --- | --- | --- | --- | --- | --- | --- | --- | --- | --- | --- | --- | --- | --- | --- | --- | --- | --- | --- | --- | --- | --- | --- | --- | --- | --- | --- | --- | --- | --- | --- | --- | --- | --- | --- | --- | --- | --- | --- | --- | --- | --- | --- | --- | --- | --- | --- | --- | --- | --- | --- | --- | --- | --- | --- | --- | --- | --- | --- | --- | --- | --- | --- | --- | --- | --- | --- | --- | --- | --- | --- | --- | --- | --- | --- | --- | --- | --- | --- | --- | --- | --- | --- | --- | --- | --- | --- | --- | --- | --- | --- | --- | --- | --- | --- | --- | --- |

| **Table S3. Drug-resistant profiles in lineage 1, lineage 2 and lineage 4 isolates.** | | | | | | | | | | | | |
| --- | --- | --- | --- | --- | --- | --- | --- | --- | --- | --- | --- | --- |
| DR pattern |  | Lineage 1 (N=16) | | |  | Lineage 2 (N=132) | | |  | Lineage 4 (N=52) | | |
|  |  | No. (%) | OR (95% CI) | *P* value |  | No. (%) | OR (95% CI) | *P* value |  | No. (%) | OR (95% CI) | *P* value |
| RIF |  | 16 (100.0) | NA | 1.000^a^ |  | 129 (97.7) | NA | 0.323^a^ |  | 52 (100.0) | NA | 0.569^a^ |
| INH |  | 13 (81.3) | 2.15 (0.59-7.83) | 0.279^a^ |  | 91 (68.9) | 1.13 (0.61-2.12) | 0.689 |  | 32 (61.5) | 0.68 (0.35-1.31) | 0.245 |
| PZA |  | 3 (18.7) | 0.92 (0.25-3.38) | 1.000^a^ |  | 27 (20.5) | 1.09 (0.52-2.28) | 0.823 |  | 10 (19.2) | 0.94 (0.42-2.08) | 0.862 |
| EMB |  | 5 (31.3) | 0.72 (0.24-2.17) | 0.560 |  | 59 (44.7) | 2.42 (1.27-4.63) | **0.007** |  | 12 (23.1) | 0.39 (0.19-0.81) | **0.010** |
| STR |  | 4 (25.0) | 0.78 (0.24-2.53) | 0.782^a^ |  | 47 (35.6) | 2.58 (1.26-5.29) | **0.007** |  | 8 (15.4) | 0.35 (0.15-0.79) | **0.009** |
| FQs |  | 1 (6.3) | 0.55 (0.07-4.36) | 0.707^a^ |  | 18 (13.6) | 3.42 (0.97-12.06) | **0.052^a^** |  | 2 (3.8) | 0.27 (0.06-1.21) | 0.111^a^ |
| KM/AMK/CM |  | 0 (0.0) | NA | 0.604^a^ |  | 10 (7.6) | 2.70 (0.58-12.71) | 0.228 |  | 2 (3.8) | 0.55 (0.12-2.61) | 0.526^a^ |
| ETO |  | 8 (50.0) | 6.08 (2.10-17.61) | **0.002** |  | 19 (14.4) | 0.59 (0.28-1.26) | 0.171 |  | 7 (13.5) | 0.70 (0.28-1.71) | 0.431 |
| PAS |  | 0 (0.0) | NA | 1.000^a^ |  | 5 (3.8) | 2.64 (0.30-23.04) | 0.438^a^ |  | 1 (1.9) | 0.56 (0.06-4.92) | 1.000^a^ |
| ^a^ Fisher’s exact probability test (two-tailed).  Abbreviations: DR, drug resistance; RIF, rifampicin; INH, isoniazid; EMB, ethambutol; PZA, pyrazinamide; STR, streptomycin; OFX, ofloxacin; MFX, moxifloxacin; KM, kanamycin; AMK, amikacin; CM, capreomycin; ETO, ethionamide; PAS, para-aminosalicylic acid; OR, odds ratio; CI, confidence interval; NA, not applicable due to a small no. of cases.  Statistical significances are represented in bold. | | | | | | | | | | | | |
